# Polymerized small molecular acceptor based all-polymer solar cells with an efficiency of 16.16% via tuning polymer blend morphology by molecular design

**DOI:** 10.1038/s41467-021-25638-9

**Published:** 2021-09-06

**Authors:** Jiaqi Du, Ke Hu, Jinyuan Zhang, Lei Meng, Jiling Yue, Indunil Angunawela, Hongping Yan, Shucheng Qin, Xiaolei Kong, Zhanjun Zhang, Bo Guan, Harald Ade, Yongfang Li

**Affiliations:** 1 Beijing National Laboratory for Molecular Sciences, CAS Key Laboratory of Organic Solids, Institute of Chemistry, Chinese Academy of Sciences, Beijing, China; 2 School of Chemical Science, University of Chinese Academy of Sciences, Beijing, China; 3 Center for Physiochemical Analysis and Measurement, Institute of Chemistry, Chinese Academy of Sciences, Beijing, China; 4 Department of Physics and Organic and Carbon Electronics Lab (ORaCEL), North Carolina State University, Raleigh, NC USA; 5 Department of Chemical Engineering, Stanford University, Stanford, CA USA; 6 Laboratory of Advanced Optoelectronic Materials, Suzhou Key Laboratory of Novel Semiconductor-Optoelectronics Materials and Devices, College of Chemistry, College of Chemistry, Chemical Engineering and Materials Science, Soochow University, Suzhou, Jiangsu China

**Keywords:** Solar cells, Solar cells

## Abstract

All-polymer solar cells (all-PSCs) based on polymerized small molecular acceptors (PSMAs) have made significant progress recently. Here, we synthesize two A-DA’D-A small molecule acceptor based PSMAs of PS-Se with benzo[c][1,2,5]thiadiazole A’-core and PN-Se with benzotriazole A’-core, for the studies of the effect of molecular structure on the photovoltaic performance of the PSMAs. The two PSMAs possess broad absorption with PN-Se showing more red-shifted absorption than PS-Se and suitable electronic energy levels for the application as polymer acceptors in the all-PSCs with PBDB-T as polymer donor. Cryogenic transmission electron microscopy visualizes the aggregation behavior of the PBDB-T donor and the PSMA in their solutions. In addition, a bicontinuous-interpenetrating network in the PBDB-T:PN-Se blend film with aggregation size of 10~20 nm is clearly observed by the photoinduced force microscopy. The desirable morphology of the PBDB-T:PN-Se active layer leads its all-PSC showing higher power conversion efficiency of 16.16%.

## Abstract (web summary)

Through development of non-fullerene acceptors, OPVs have reached efficiencies of 18%, yet the inadequate operational lifetime still poses a challenge for the commercialisation. Here, the authors investigate the origin of instability of NFA solar cells, and propose some strategies to mitigate this issue.

## Introduction

All-polymer solar cells (all-PSCs), based on the bulk heterojunction (BHJ) active layers composed of a *p*-type conjugated polymer donor and an *n*-type conjugated polymer acceptor, have attracted growing attentions due to their advantages of good mechanical properties, photostability, and thermal stability compared with small molecule acceptor (SMA)-based organic solar cells^1–5^. Recently, benefitted from the concept of polymerized small molecule acceptors (PSMAs)^6,7^, the power conversion efficiency (PCE) of the all-PSCs based on PSMA increases rapidly^8–10^, which has reached the threshold for commercialization and some specific applications like wearable and flexible devices^11,12^.

To establish high-performance all-PSCs, highly efficient polymer acceptor materials and the proper morphology of photoactive films are indispensable. In the development of polymer acceptors, an important breakthrough is the design strategy of PSMAs proposed by our group in 2017^6^, which is composed of a narrow bandgap SMA as main building block polymerized with a π-bridge linking unit. The PSMAs preserve the advantages of SMAs (such as narrow bandgap, strong absorption coefficient, and suitable electronic energy levels) and the advantages of polymers (such as higher stability and better flexibility) simultaneously^7^. The first PSMA PZ1 based on polymerized IDIC-C16 realized a PCE of 9.19% for the all-PSCs in 2017^6^, which was the highest PCE of the all-PSCs at that time. Then, by using PM6 as the polymer donor, the PCE of the PZ1-based all-PSCs reached 11.2%^13^. After the A-DA’D-A structured SMA Y6 was reported in 2019^14,15^, the PSMAs based on the A-DA’D-A type SMA units have rapidly boosted the PCE of the all-PSCs up to over 15%^16–18^, owing to their narrower bandgap and smaller voltage loss. Thus, the selection of the small molecule acceptor building blocks plays a significant role in the photovoltaic properties of the PSMAs.

Moreover, a well-formed morphology of the all-polymer active layer with bicontinuous-interpenetrating network of polymer donor/polymer acceptor is essential to efficient exciton dissociation and charge carrier transport. In the PSCs based on small molecule acceptors, the strategies widely adopted for adjusting morphology involve addition of solvent additive, treatments of solvent annealing and thermal annealing, and processing with hot spin coating, etc^19–21^. However, compared with the blend films based on polymer donors and SMAs, most of the above-mentioned methods for morphological control are not particularly effective in the all-PSCs, because both the polymer donor and polymer acceptor possess long molecular skeleton and stronger intermolecular interaction^22^, which often leads to preaggregation of the polymers in precursor blend solutions and becomes less sensitive to post-treatments of the polymer films. For example, the morphologies of the PSMAs of PJ^23^ and PTPBT-ET_x_^24^ exhibit insensitivity to the thermal annealing temperature or the volume ratio of solvent additives treatment in a certain range. Since intensive researches have revealed the interplay between the molecular structures and the blend film morphologies of the polymer donor and SMAs^25,26^, therefore the proper selection of SMA building blocks is critical in the design of PSMAs for modulating its electronic properties and adjusting its aggregation morphology.

As discussed above, PSMAs with suitable aggregation and good solubility should be developed for realizing high-performance all-PSCs. So far, however, most of the latest reported PSMAs were based on the A-DA’D-A-structured Y6-like building block containing benzo[c][1,2,5]thiadiazole (BS) A’-core in their DA’D fused ring. Earlier studies have illustrated that the Y6 molecule has strong intermolecular packing^27^, and may cause difficulty in the balance of aggregation and solubility for the PSMAs. To address this issue, we consider to switch the building block to Y18-like unit containing benzotriazole (BN) A’-core^28^ with an additional alkyl side chain on the top nitrogen of BN core, and Y18 is developed by our group in collaboration with Zou’s group recently^28^. The one more alkyl side chain on the Y18-like unit (totally five side chains) could provide more possibilities of adjusting the aggregation and solubility, and could facilitate the modulation of the film morphology. Moreover, Y18 shows a slightly narrower bandgap than Y6, while the PM6:Y18 based PSCs display a higher open-circuit voltage (*V*_oc_) than that of the PM6:Y6 based devices benefitted from its lower energy loss^28^. The advantages of Y18 on the tunable aggregation, broad absorption and lower energy loss could be brought into its corresponding PSMAs^18^.

**Fig. 1:**
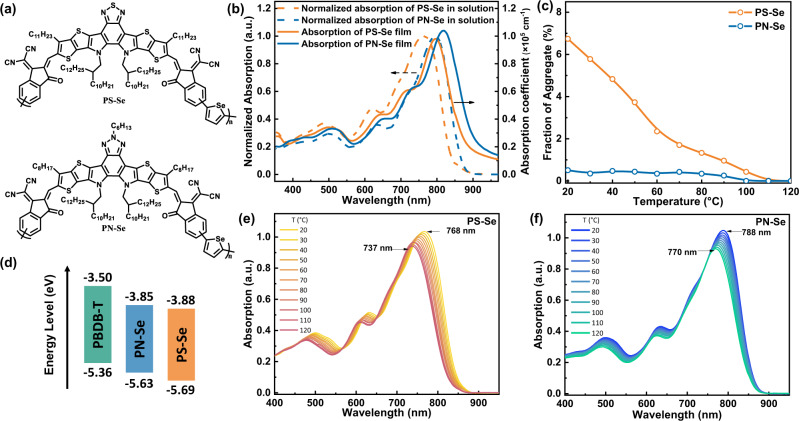
Molecular structures and physicochemical properties of PS-Se and PN-Se. **a** Chemical structures of PS-Se and PN-Se acceptors. **b** UV-vis absorption spectra of PS-Se and PN-Se in chloroform solutions and the corresponding neat films. **c** Plots of calculated aggregate content (molar %) of the two polymer acceptors in chlorobenzene at different temperatures. **d** Energy level diagram of the related materials in the all-PSCs. Variable-temperature UV-vis absorption spectra of (**e**) PS-Se and (**f**) PN-Se in chlorobenzene.

Based on above considerations, in this work, we designed and synthesized a new PSMA PN-Se (see Fig. 1a) with the A-DA’D-A type Y18-like unit (with BN A’-core) as main building block and selenophene as π-linking units in considering the red-shifted absorption of selenophene^29^. And we synthesized an analogue PSMA PS-Se (see Fig. 1a) with the Y6-like unit (with BS A’-core) as the main building block, for the studies of the A’-core structure on the photovoltaic performance of the PSMAs. As mentioned above, the BN unit in PN-Se has one more alkyl side chain compared with the BS unit in PS-Se, which could affect the molecular packing of the corresponding polymer acceptors and the morphology of the blend film of the polymer acceptors with polymer donor. We compared the aggregation behavior of the two PSMAs by variable-temperature UV-vis absorption spectroscopy and by Cryogenic transmission electron microscopy (Cryo-TEM) in the solutions. In the all-PSCs, the preaggregated microstructure of the polymer donor and polymer acceptor in their blend precursor solution is generally considered to dominate the active layer film morphology^30–32^. However, there is no report on direct observation or study of these preaggregation state of the polymers blend in their solutions till now. Cryo-TEM is a technique to visualize the aggregation behavior of the molecules in solution by measuring the TEM morphology of the frozen solution sample^33,34^. Here, for the first time, we measured the morphology of the polymer micro-aggregates in the rapidly frozen blend precursor solutions of polymer donor and PSMAs by the Cryo-TEM. In addition, photo-induced force microscopy (PiFM)^35^ was employed to characterize the morphology of the polymer blend films and to distinguish the phase region of the donor and acceptor polymers. Based on these measurements, we found that the PSMA PN-Se polymer acceptor exhibited more suitable phase separation and red-shifted absorption. The all-PSC based on PN-Se as polymer acceptor and PBDB-T^36^ as polymer donor demonstrated a high PCE of 16.16% with a *V*_*oc*_ of 0.907 V, *J*_*sc*_ of 24.82 mA cm^−2^, and FF of 0.718, while the PS-Se-based all-PSC showed a moderate PCE of 13.83% with a *V*_*oc*_ of 0.874 V, *J*_*sc*_ of 23.27 mA cm^−2^, and fill factor (FF) of 0.680. The better photovoltaic performance of the PN-Se based device is benefitted from its more suitable phase separation, faster exciton dissociation and charge transportation and less charge recombination. These results demonstrate the importance of selecting suitable SMA building block unit in the design of high-performance PSMAs, and provide an effective way to adjust the photoactive layer morphology in the all-PSCs.

## Results

### The synthesis of PSMAs PS-Se and PN-Se

**Fig. 2:**
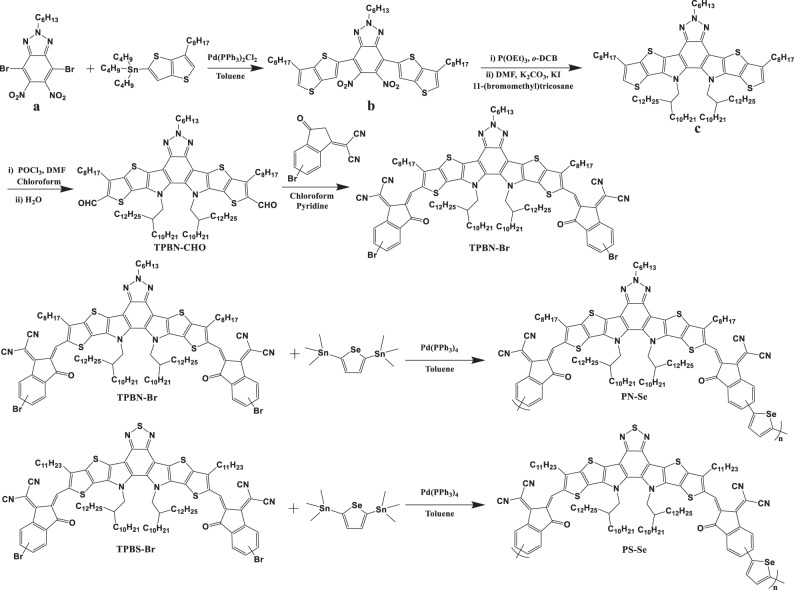
Synthetic routes of the PSMAs. The synthetic routes of PS-Se and PN-Se.

Figure 2 shows the chemical structures and the synthetic routes of PS-Se and PN-Se. Both PSMAs were synthesized by Stille cross-coupling polymerization of the brominated Y6-like TPBS-Br unit or brominated Y18-like TPBN-Br unit and the 2,5-bis (trimethylstannyl) selenophene linking bridge-unit (see synthetic details in the “Methods” and “Supplementary methods” sections). To ensure the solubility of the PSMAs PS-Se and PN-Se, longer alkyl side chains were employed in the SMA units. Thus, the resulting polymers can be readily dissolved in common organic solvents at room temperature. In order to reduce the influence of the molecular weights of the polymer acceptors on their physicochemical and photovoltaic properties, two batches of PS-Se and PN-Se were selected with comparable number-average molecular weights (*M*_*n*_) measured by gel permeation chromatography (GPC), as shown in Supplementary Fig 19. The number-average molecular weight and polydispersity index were 11.5 kDa/2.7 and 11.7 kDa/2.8 for PS-Se and PN-Se, respectively. Thermal stabilities of PS-Se and PN-Se were characterized by thermogravimetric analysis (TGA), and both PSMAs showed good thermal stability with decomposition temperature (*T*_d_, 5% weight loss) at 343 °C under nitrogen atmosphere (as shown in Supplementary Fig. 1).

### Absorption spectra and electronic energy levels of the PSMAs

UV-vis absorption spectra of PS-Se and PN-Se in chloroform solution and neat films were measured to investigate the effect of the BS and BN core on the optical and packing properties of the PSMAs. As shown in Fig. 1b, either in chloroform solution or film state, PN-Se shows more red-shifted absorption than PS-Se. In solution, PS-Se and PN-Se both exhibit broadened absorption range from 600 to 900 nm with maximum peaks at 766 and 796 nm, respectively. In neat films, PS-Se displays an absorption peak at 798 nm with absorption coefficient of 0.98 × 10^5^ cm^−1^, and PN-Se shows a featured peak at 820 nm with absorption coefficient of 1.04 × 10^5^ cm^−1^. It is worth noting that the degree of red-shift for PS-Se and PN-Se films were observed as 32 and 24 nm, respectively, in comparison with its solution. The larger degree of red-shift is associated with more intimate π–π stacking interaction and aggregation^37^. Since the changes in absorption range and intensity are the signature of aggregation of the polymers, variable-temperature UV-vis absorption spectra were further measured in the temperature range of 120–20 °C (as shown in Fig. 1e, f). At the high temperature of 120 °C, the polymer chains will be in disordered phase. During the cooling process from the high temperature of 120 °C, a fraction of the polymer chains will be aligned in a well-ordered aggregation while the rest of polymer chains remain disordered^38^, which could affect the overall absorption spectra. This fraction of aggregation (*f*_*aggr*_) could be calculated according to the change between the absorption of the pure disordered phase at high temperature (120 °C) and the superposition absorption of disordered and aggregated chains at the lower temperature^39^ (Fig. 1c). From 120 to 20 °C, the PS-Se film exhibits more red-shifted vibronic absorption peak (by 31 nm from 737 to 768 nm) than that of the PN-Se film (by 18 nm from 770 to 788 nm), which should be associated with the stronger aggregation in PS-Se. While the smaller red-shift in vibronic feature of the PN-Se film might also be caused by planarization of the disordered chains rather than aggregation^38^.

Frontier orbital energy levels (HOMO/LUMO) of the PS-Se and PN-Se were estimated from electrochemical cyclic voltammetry (CV) measurements (Fig. 1d). From the onset oxidation potential (*E*_ox_) and onset reduction potential (*E*_red_), the HOMO energy level (*E*_HOMO_) and the LUMO energy level (*E*_LUMO_) of the polymers were calculated according to the equation *E*_LUMO/HOMO_ = −e (*E*_red/ox_ + 4.36) (eV) where the unit of *E*_red/ox_ is V vs. Ag/AgCl. Redox potential of Fc/Fc^+^ is 0.44 V vs. Ag/AgCl in our measurement system (see Supplementary Fig. 2), and we took the energy level of Fc/Fc^+^ as 4.8 eV below vacuum. The *E*_LUMO_/*E*_HOMO_ values were estimated to be −3.88/−5.69 eV for PS-Se, and −3.85/−5.63 eV for PN-Se, respectively. The electronic energy levels and other physicochemical properties of the polymer acceptors are listed in Supplementary Table 1 for a clear comparison.

### Visualization of aggregation behavior of the polymer blends in solution by Cryo-TEM

**Fig. 3:**
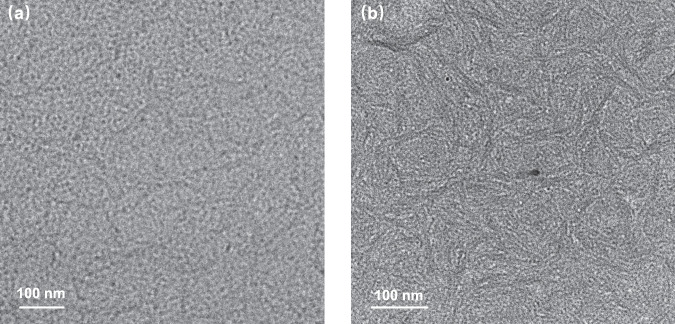
Cryo-TEM images. **a** Cryo-TEM images of the frozen PBDB-T:PN-Se blend precursor solution in chloroform. **b** Cryo-TEM images of the frozen PBDB-T:PS-Se blend precursor solution in chloroform.

Cryo-TEM is a unique technique to characterize the aggregation and dispersion structures dispersed in liquid suspensions that are close to their native state^33,40,41^. After the rapid freezing of the polymer donor/polymer acceptor blend precursor solutions, the preaggregation state dispersed in the solution could be visualized and characterized by high-resolution TEM. As mentioned above, the morphology of the polymer donor/polymer acceptor blend films is mainly controlled by the preaggregation state in their solutions. Hence, we performed the Cryo-TEM measurements to visualize the preaggregation state of the polymer blends in their chloroform solutions. In this work, PBDB-T was used as the polymer donor, and the concentration of 14 mg mL^−1^ for the total polymer donor and acceptor was adopted in the all-polymer blend solutions (see sample preparation details in “Method” section). Figure 3 shows Cryo-TEM images of the frozen polymer blend chloroform solutions. In the frozen PBDB-T:PN-Se blend solution, the polymers show dark gray thin lines with a uniform aggregation size of ca. 8–16 nm (see Fig. 3a), which could form the ideal interpenetrating networks morphology in the BHJ blend films prepared from the solution. While for the PBDB-T:PS-Se blend solution (see Fig. 3b), larger dense blocks of dispersed aggregates were observed, and the reticular aggregates with slightly larger size of ca. 14–23 nm were more coarsely distributed. The results of the Cryo-TEM indicate that the chemical structures of PSMA plays an important role on the aggregation morphology in the blend solutions. Obviously, the different sizes and shapes of the aggregates of the polymer blends in their solutions would affect the final film morphology prepared from the solution when fabricating the all-PSCs.

### Morphological characterizations of polymer films

First, the Flory-Huggins interaction parameters (*χ*_da_) were calculated from contact-angle measurements of the neat films. The corresponding surface energy of the polymer donor (*γ*_d_) and acceptors (*γ*_a_) (Supplementary Table 2) could assess the miscibility between the donor and acceptor, according to the equation ${\chi }_{{{{{{\rm{da}}}}}}}=K{\left(\sqrt{{\gamma }_{d}}-\sqrt{{\gamma }_{{{{{{\rm{a}}}}}}}}\right)}^{2}$, where *K* is a constant^42^. The *χ*_da_ values were 0.30 *K* and 0.19 *K* for PBDB-T:PS-Se and PBDB-T:PN-Se blend films, respectively. The smaller *χ*_da_ of PBDB-T:PN-Se suggests greater miscibility and is correlated with the increasing density of D/A interfaces in the blend films^43^.

**Fig. 4:**
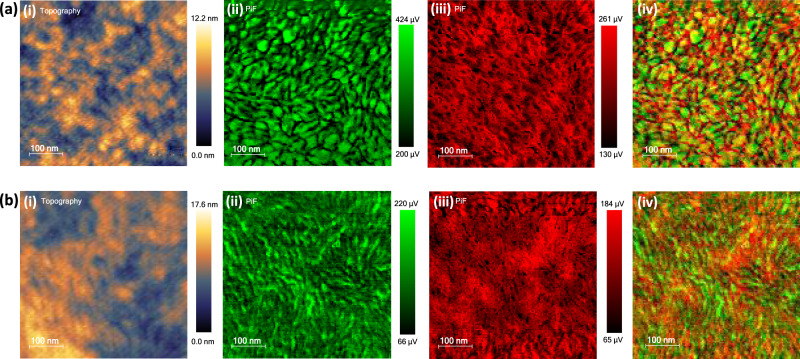
AFM and PiFM images of the blend films. **a** Patterns of PBDB-T:PN-Se blend. **b** Patterns of PBDB-T:PS-Se blend. (i) AFM topography images of corresponding blend films. (ii) PiFM images of PBDB-T. (iii) PiFM images of the PSMA. (iv) Combined images of (ii) and (iii) to provide chemical map of PBDB-T and PSMA.

To further investigate the effect of BS core in PS-Se and BN core in PN-Se on the morphological characteristics, the morphologies of the PS-Se- and PN-Se-based blend films were measured by atomic force microscopy (AFM) and photo-induced force microscopy (PiFM). Combining the response of photo-induced polarizability of polymer donor or polymer acceptor in the near field and AFM technology, PiFM is capable of distinguishing the donor and acceptor at nanometer scale and providing a thorough and direct compositional analysis of the all-polymer BHJ films^35,44^. First, we measured infrared (IR) absorption spectra of neat PS-Se and PN-Se films (see the Supplementary Fig. 3), and both films presented several IR characteristic peaks. Here, the IR absorption wavenumber of 1653 cm^−1^ was chosen as the characteristic peak of donor PBDB-T component and 1538 cm^−1^ as the characteristic peak of acceptor PS-Se or PN-Se component. Figure 4 shows the AFM and PiFM images of the PBDB-T:PS-Se and PBDB-T:PN-Se blend films which are fabricated by the same optimized conditions in their corresponding all-PSCs. As shown in Fig. 4a(i), the AFM topography pattern of PBDB-T:PN-Se blend film displays distinct and uniform phase separation with small fibrous aggregates. In particular, the PiFM images of PBDB-T (green color, as shown in Fig. 4a(ii)) and PN-Se (red color, as shown in Fig. 4a(iii)) all show more distinct fibrous network. After combining the PiFM images of PBDB-T and PN-Se in Fig. 4a(iv), the blend film exhibits an unique BHJ bicontinuous-interpenetrating network of polymer donor/polymer acceptor with PN-Se polymer acceptor surrounding the PBDB-T polymer donor region. The domain size of the distinguishable phase separation was estimated to be 10–20 nm, which is beneficial for exciton dissociation and charge transportation^45^ and could lead to higher photovoltaic performance of the all-PSCs. In contrast, PBDB-T:PS-Se blend film (Fig. 4b(iv)) shows bigger donor domain (green color) and acceptor domain (red color) with the oversized phase separation over 30 nm of PBDB-T and PS-Se, which is consistent with the stronger aggregation of PS-Se confirmed from the variable-temperature UV-vis absorption and the Cryo-TEM analysis mentioned above. In general, the ideal phase separation scale should be around 20 nm^46^. The oversized phase separation decreases the interface areas in the BHJ films, which has negative effect on exciton dissociation and charge separation, and would influence the photovoltaic performance^11^.

Similarly, the AFM topography image (Fig. 4a(i)) of PBDB-T:PN-Se shows smoother surface with smaller values of root-mean-square (RMS) roughness of 1.67 nm, compared with the PBDB-T:PS-Se blend film (Fig. 4b(i)) with RMS of 2.21 nm. The smoother surface could favorably improve the contact between the interfacial layer and the active layer^47^. Besides, the TEM images, as shown in Supplementary Fig. 4, exhibit similar phase separation tendency to the PiFM images.

**Fig. 5:**
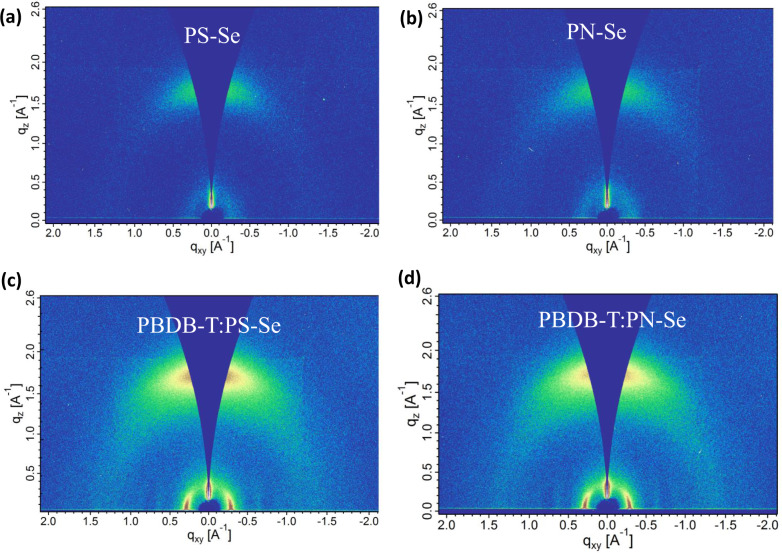
2D-GIWAXS scattering patterns. 2D-GIWAXS scattering patterns of PS-Se and PN-Se neat films (**a**, **b**), and their blend films (**c**, **d**) with polymer donor PBDB-T.

In addition to phase separation size, the crystallinity and polymer chain arrangement also influence the exciton diffusion, exciton dissociation and free charge carrier transport. Thus, we further conducted grazing-incidence wide-angle X-ray scattering (GIWAXS) measurements^48^. Figure 5a, b shows the two-dimensional (2D) patterns and Supplementary Fig. 5 a, b shows the one-dimensional (1D) extracted profiles of the neat films. PS-Se and PN-Se neat films display clear (010) peaks in out-of-plane (OOP) direction and (100) peak in in-plane (IP) direction, suggesting that both acceptors contain preferred face-on orientations. Comparatively, PS-Se shows (010) peak at *q* = 1.65 Å^−1^ (*d*_(010)_ = 3.81 Å) while PN-Se at *q* = 1.66 Å^−1^ (*d*_(010)_ = 3.78 Å), and the corresponding crystal coherence lengths (CCLs) of (010) orientation are 19.1 and 16.5 Å calculated by Scherrer equation for PS-Se and PN-Se, respectively. Besides, for the blend films, the 2D-GIWAXS scattering patterns and the corresponding 1D in- and out-of-plane sector cuts are shown in Fig. 5c, d and Supplementary Fig. 5 c, d, respectively, and the relevant molecular packing parameters are summarized in Supplementary Table 3 and 4. Overall, all the blend films maintain the clear face-on orientation. To better understand the differences between PBDB-T:PS-Se and PBDB-T:PN-Se, the molecular packing and CCLs of the individual donor and acceptor in the blends were investigated by deconvoluting the (010) and (100) peaks to its individual donor and acceptor components. For the donor PBDB-T, the CCL value of the (010) peak in the PBDB-T:PS-Se blend film is 28.6 Å, which is slightly larger than 27.3 Å in the PBDB-T:PN-Se blend film. In general, longer (010) CCL implies better molecular ordering along the π–π packing orientation of the PBDB-T:PS-Se film. On the other hand, for the polymer acceptor, both PBDB-T:PS-Se and PBDB-T:PN-Se blend films show similar (100) peak at *q* = 0.28 Å^−1^ (*d*_(100)_ = 22.4 Å CCL = 31.4 Å) and (010) peak at *q* = 1.65 Å^−1^ (*d*_(010)_ = 3.81 Å CCL = 11.6 Å), indicating that there is no much difference of the π–π stacking distances and the coherences across the films between the two polymer blend active layers. However, the integrated intensity (normalized to the thickness (Volume) and the background) of the π–π stacking (010) peak and lamellar stacking (100) peak are marginally higher for both the acceptor and the donor components of the PBDB-T:PN-Se blend, suggesting a favorably oriented and more ordered arrangement of the PN-Se and PBDB-T grains for its better charge transport properties. The relatively higher diffracted intensity can be interpreted as a smaller volume fraction of disordered material^49^. Additionally, the corresponding *g*-parameters^50^ were calculated for PBDB-T:PS-Se (*g* = 14.3 for donor and *g* = 22.8 for acceptor) and PBDB-T:PN-Se (*g* = 14.6 for donor and 22.8 for acceptor), indicating the material is amorphous in the π–π stacking direction of the polymers^51^. In this case, the ordered arrangement of the aggregates and presence of interconnected morphology such as better bicontinuous-interpenetrating polymer donor/polymer acceptor networks are more important for charge separation and transportation.

### Photovoltaic performance of the all-PSCs

**Fig. 6:**
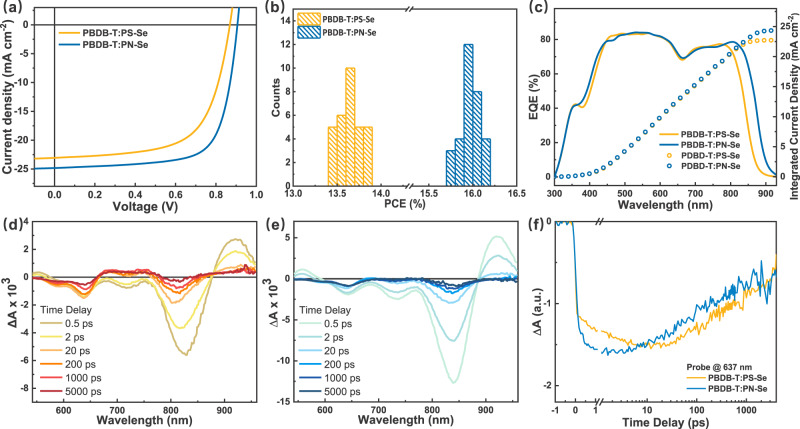
Photovoltaic performance and transient absorption kinetics. **a** The *J–V* curves of the optimal all-PSCs under the illumination of AM1.5 G, 100 mW cm^−2^. **b** Histogram of PCE measurement for 30 devices based on PBDB-T:PS-Se (1.5:1, w/w) and 30 devices based on PBDB-T:PN-Se (1.5:1, w/w), measured under the illumination of AM1.5 G, 100 mW cm^−2^. **c** External quantum efficiency (EQE) spectra of the corresponding all-PSCs. Femtosecond transient absorption spectra of (**d**) PBDB-T:PS-Se and (**e**) PBDB-T:PN-Se with excitation at 850 nm. **f** Transient kinetic traces of PBDB-T GSB probing at 637 nm for the blend films.

The photovoltaic performance of the PSMA polymer acceptors were studied by fabricating the all-PSCs with the conventional device structure of ITO/PEDOT:PSS/PBDB-T:PSMA/PDINN^52^/Ag. The donor to acceptor weight ratios were fixed at the optimized weight ratio of 1.5:1, and the current density–voltage (*J*–*V*) curves of the all-PSCs were measured under the illumination of AM1.5 G, 100 mW cm^−2^. Detailed processes of fabricating the all-PSCs are described in “Method” section. Figure 6a shows the optimal *J*–*V* curves and Table 1 lists the photovoltaic parameters of the devices. The all-PSC based on PBDB-T:PS-Se showed a PCE of 13.83% with a *V*_*oc*_ of 0.874 V, *J*_*sc*_ of 23.27 mA cm^−2^, and FF of 0.680. As expected, the PBDB-T:PN-Se based all-PSC achieved an even better PCE of 16.16% with a *V*_*oc*_ of 0.907 V, *J*_*sc*_ of 24.82 mA cm^−2^, and FF of 0.718. In comparison with the PS-Se-based device, the accountable reasons for the higher PCE of the PN-Se-based all-PSC are the further increased *V*_*oc*_ benefited from the higher lying LUMO energy level of the PN-Se polymer acceptor, the enhanced *J*_*sc*_ resulted from the red-shifted absorption and higher absorption coefficient of PN-Se, and the better FF benefitted from the well-formed blend morphology of the PBDB-T:PN-Se blend active layer. Figure 6b further shows the PCE histogram obtained from the PBDB-T:PS-Se- and PBDB-T:PN-Se-based devices, indicating the better reproducibility of the high photovoltaic performance of the PBDB-T:PN-Se-based all-PSCs. In addition, Supplementary Table 7 lists the molecular weights of PS-Se and PN-Se and the PCEs of the all-PSCs with PBDB-T polymer donor and the PSMA polymer acceptors with different molecular weights. It can be seen that for the different batches of PSMA acceptors in the molecular weight range of 8–12 kDa, all the PN-Se-based all-PSCs show higher PCE than that of the PS-Se-based devices.

**Table 1:** Photovoltaic parameters of the optimal all-PSCs based on PBDB-T:PSMAs (1.5:1, w/w), measured under the illumination of AM1.5 G, 100 mW cm^−2^.

Active layer	*V*_oc_ (V)	*J*_sc_ (mA cm^−2^)	FF	PCE (%)
PBDB-T:PS-Se	0.874 (0.874 ± 0.003)	23.27 (22.84 ± 0.37)	0.680 (0.693 ± 0.009)	13.83 (13.64 ± 0.12)^a^
PBDB-T:PN-Se	0.907 (0.907 ± 0.003)	24.82 (24.73 ± 0.26)	0.718 (0.713 ± 0.005)	16.16 (15.97 ± 0.13)^a^

^a^The average parameters were calculated from over 30 devices.

The external quantum efficiency (EQE) of the PBDB-T:PS-Se- and PBDB-T:PN-Se-based devices were measured and shown in Fig. 6c. Both all-PSC devices show broad and stronger photoresponse from 300 to 900 nm, while the PN-Se-based all-PSC displayed a broader photoresponse extending to over 900 nm, benefitted from the red-shifted absorption of PN-Se. The *J*_sc_ values integrated from the EQE values are 22.72 and 24.36 mA cm^−2^ for the PS-Se- and PN-Se-based devices, respectively. Both the integrated *J*_sc_ values are in good agreement with those values obtained from the *J–V* characteristics of the all- PSCs.

Thermal stability is one of the major advantages of all-PSCs. We examined the thermal stability of the active layer films based on PS-Se and PN-Se. The active layers were annealed at 150 °C for different times before fabricating into all-PSC devices. After 600 min of thermal annealing stress, photovoltaic performance of the PS-Se and PN-Se based all-PSCs did not show significant degradation (as shown in Supplementary Fig. 6). The results prove that the blend films based on PS-Se and PN-Se have good thermal stability under the harsh test conditions.

We further carried out the studies on the photo-induced charge transfer (CT) process of the active layer by measuring the femtosecond transient absorption spectra (fs-TA) of the blend films of PBDB-T:PS-Se and PBDB-T:PN-Se^53^. Pump wavelength was set to 850 nm to selectively excite the polymer acceptors in the blend films. Figure 6d shows the fs-TA spectra of the PBDB-T:PS-Se blend at the selected time delays. Photoexcitation of the acceptor leads to the formation of PS-Se excitons in the film, a broad ground state bleach (GSB) peak at 825 nm was observed that matches the absorption spectrum of PS-Se. Additional GSB was also apparent at 637 nm, which is attributed to the donor PBDB-T, resulted from the ultrafast hole transfer from PS-Se to PBDB-T directly at the D/A interface. With the decay of PS-Se excited state, the intensity of the GSB of PBDB-T gradually grows over the subsequent 20 ps, indicating a continuous hole transfer process. The GSB of PS-Se and PBDB-T then decays concomitantly, since the CT state starts to recombine to ground state. Similar transient absorption spectra were also observed for the PBDB-T:PN-Se blend except for the red-shifted GSB of PN-Se (Fig. 6e). The results agree well with the high EQE values of both all-PSCs based on PBDB-T:PS-Se and PBDB-T:PN-Se.

Then we monitored the kinetic trace of the GSB of PBDB-T for both blends, the hole transfer dynamics at 637 nm were extracted in Fig. 6f. The PN-Se based blend exhibited a faster hole transfer rate than that of PS-Se. We further investigated the carrier transport properties by measuring the charge carriers mobility with the space-charge-limited-current (SCLC) method. As shown in Supplementary Fig. 7 and Supplementary Table 8, the average electron mobilities of ten devices based on PBDB-T:PS-Se and PBDB-T:PN-Se were estimated to be (4.84 ± 0.48) × 10^−4^ and (6.87 ± 0.50) × 10^−4^ cm^2^ V^−1^ s^−1^, respectively. And the average hole mobilities of ten devices based on PBDB-T:PS-Se and PBDB-T:PN-Se were estimated to be (6.89 ± 0.52) × 10^−4^ and (7.92 ± 0.49) × 10^−4^ cm^2^ V^−1^ s^−1^, respectively. The electron and hole mobilities of the PN-Se-based blend film are higher than those of the PS-Se-based blend film. For the PN-Se-based blend, its higher hole mobility agrees with the hole transfer dynamics results shown in Fig. 6f and could be benefitted from the ideal phase separation and better D/A interpenetrating structure in its blend films as mentioned above. Moreover, the smaller *μ*_h_/*μ*_e_ ratio (1.15) in the PBDB-T:PN-Se blend film indicates more balanced electron/hole transport capability of the PBDB-T:PN-Se blend film. The higher and more balanced electron/hole mobilities could be responsible to the higher FF of the PN-Se based all-PSCs^54^.

To further evaluate the carrier trapping and detrapping in the all-polymer blend, we measured the transient photocurrent (TPC). The photocurrent curves of the PS-Se- and PN-Se-based devices all displayed little change with light intensity up to 1 sun equivalent (as shown in Supplementary Fig. 8a, b). In details, as the light intensity increasing from 0.1 to 1 sun, the decay times shortened from 0.40 to 0.34 μs for the PS-Se based devices and from 0.34 to 0.25 μs for the PN-Se-based devices (see Supplementary Fig. 8c). In general, the faster rise/fall behavior of photocurrent and shorter transport time (*τ*_t_) indicate favorable charge carrier transport, less electron trap states and faster transport-limiting recombination^55^. Furthermore, transient photovoltage (TPV) decays were measured to estimate the charge carrier lifetimes (*τ*). The carrier lifetimes of the PN-Se based device are consistently longer than the PS-Se-based device across the entire light intensity range from 0.1 to 1 sun (as shown in Supplementary Fig. 8d), which indicates less carrier recombination^46^. All the results mentioned above are matched well with the morphology analysis and the excellent photovoltaic performance of the PN-Se-based all-PSCs.

## Discussion

In this work, we designed and synthesized two new PSMAs PS-Se and PN-Se, based on A-DA’D-A structured Y6-like SMA with benzo[c][1,2,5]thiadiazole (BS) A’-core or Y18-like SMA with benzotriazole (BN) A’-core as building blocks, polymerized with selenophene as π-bridge linking unit. The analysis from Variable-temperature UV-vis absorption and Flory-Huggins interaction parameters (*χ*_da_) indicate that the PN-Se shows suitable aggregation and better miscibility with PBDB-T polymer donor, compared to PS-Se, leading to the desirable phase separation in the polymer blend film. More importantly, the Cryo-TEM images of the frozen polymer blend solutions confirm the ideal polymer donor/polymer acceptor interpenetrating networks with phase separation size of ca. 8–16 nm in the preaggregation state of the PBDB-T:PN-Se blend solution. The results of PiFM analysis further clearly reveal that the PBDB-T:PN-Se blend exhibits distinct bicontinuous-interpenetrating polymer donor/polymer acceptor networks, which improves the exciton dissociation and charge transportation in the all-PSC. In addition, GIWAXS results indicate a more ordered and popularly oriented arrangement of the PN-Se-based blend film than that of PS-Se. Eventually, the PBDB-T:PN-Se-based all-PSC demonstrates the higher PCE of 16.16%, with a preferable *V*_*oc*_ of 0.907 V, *J*_*sc*_ of 24.82 mA cm^−2^, and FF of 0.718. This work not only realizes a highly efficient all-PSC based on a high-performance polymer acceptor PN-Se, but also provides a clear understanding of the relationship among the molecular structure, nanoscale morphology, photophysical properties, and photovoltaic performance, which could provide insights for the future molecular design of PSMAs and device optimization of the all-PSCs.

## Methods

### Materials and synthesis

All chemicals and solvents were purchased from Innochem, J&K, Alfa Aesar, and TCI Chemical Co., respectively. Other common materials and chemical reagents were purchased from commercial sources and used as received. Toluene was distilled from sodium and benzophenone under nitrogen before using. Polymer donor PBDB-T was purchased from Solarmer Materials Inc., and TPBS-Br was purchased from Hyper Inc.

The synthetic routes of the conjugated polymer acceptors PS-Se and PN-Se are shown in Fig. 2. The precursor **a** was synthesized by a simple nitration of 4,7-dibromo-2-hexyl-2H-benzo[d][1,2,3]triazole. The detailed synthetic procedures and characterizations of the chemical structures of the other monomers and the polymer acceptors are described in the Supplementary method section. The NMR spectra of the compounds are included in Supplementary Fig. 9~18.

### Material characterization

^1^H-NMR and ^13^C-NMR spectra were measured on a Bruker DMX-400 spectrometer with *d*-chloroform as the solvent and trimethylsilane as the internal reference. While for the PSMA PS-Se and PN-Se, ^1^H-NMR spectra were measured with 1,1,2,2,-Tetrachlorethane-D2 as solvent at 80 °C. Mass spectra measurement was performed on a Shimadzu spectrometer. TGA was conducted under a nitrogen flow rate of 100 mL min^−1^ on a Perkin-Elmer TGA-7 thermogravimetric analyzer at a heating rate of 20 °C min^−1^. UV–visible absorption spectra and Variable-temperature UV-vis absorption spectra were measured on a Hitachi U-3010 UV-vis spectrophotometer. The electrochemical cyclic voltammetry was carried out on a Zahner IM6e Electrochemical Workstation, in an acetonitrile solution of 0.1 mol L^−1^ *n*-Bu_4_NPF_6_ at a potential scan rate of 100 mV s^−1^. The sample film on Pt plate was used as working electrode, a platinum wire was used as counter electrode and Ag/AgCl was used as reference electrode. The ferrocene/ferrocenium (Fc/Fc^+^) couple was used as an internal reference. Gel permeation chromatography (GPC) measurement was performed on Agilent PL-GPC 220 instrument with high temperature chromatograph, using 1,2,4-trichlorobenzene as the eluent at 160 °C.

### Cryo-TEM measurements

The samples for the Cryo-TEM observation were prepared with the Vitrobot Mark IV (ThermoFisher Scientific). Before the preparation of samples, we put the grid in the Vitrobot Mark IV at room temperature, which is the same temperature as that of the glove box where we fabricate the all-PSCs devices. After the Vitrobot Mark IV maintaining at room temperature (rt) for 30 min, 3 μl chloroform solution dispersion (14 mg ml^−1^, rt) was dripped onto a holey carbon film on copper grid (GIG) and blotted with a piece of filter paper to obtain a thin liquid film on the grid. Then the grid was quickly plunged into liquid ethane. (At this time, the morphology of the polymer donor and PSMA had been frozen.) The vitrified samples were then transferred to a 626 cryogenic sample holder (Gatan) and examined with a Cryo-TEM (Themis 300, ThermoFisher Scientific) at 77 K. Micrographs were captured with a Falcon III camera (ThermoFisher Scientific).

### Device fabrication and characterization

The all-polymer solar cells were fabricated with a structure of ITO/PEDOT:PSS (40 nm)/active layer/PDINN/Ag. A thin layer of PEDOT:PSS was deposited on precleaned ITO-coated glass through spin coating a PEDOT:PSS aqueous solution (Baytron P VP AI 4083 from H.C. Starck) at 4000 rpm and dried subsequently at 150 °C for 15 min in air, then the PEDOT:PSS-coated ITO glass was transferred to a nitrogen glove box. The PBDB-T:polymer acceptors (1.5:1, w/w) were dissolved in chloroform (the total concentration of blend solutions was 14 mg mL^−1^ for all blends), with the addition of 1.5 vol % 1-chloronaphthalene (CN) as additive, and stirred overnight in a nitrogen-filled glove box. The blend solutions were spin-cast onto the PEDOT:PSS layer at a spin-coating rate of 3500 rpm for 30 seconds. After the active layers were treated with thermal annealing, the methanol solution of PDINN with a concentration of 1.0 mg mL^−1^ was spin-coated atop the active layer at 3000 rpm for 30 seconds to form a cathode buffer layer. Finally, top Ag electrode was deposited in vacuum onto the cathode buffer layer under high vacuum. The active area of the PSCs was 5.0 mm^2^, which was defined by Optical microscope (Olympus BX51). In order to accurately measure the photocurrent, mask with an area of 4.80 mm^2^ was used to define the effective area of the OSCs. The devices with or without mask showed consistent photovoltaic performance values with relative errors within 0.3%

The current density–voltage (*J–V*) curves of the all-PSCs were measured on Keithley 2450 Source-Measure Unit in a glove box filled with nitrogen (oxygen and water contents are smaller than 0.1 ppm). And the measurements were performed by scanning voltage from −1.5 to 2 V with a voltage step of 10 mV and delay time of 1 ms. Oriel Sol3A Class AAA Solar Simulator (model, Newport 94023 A) with a 450 W xenon lamp and an air mass (AM) 1.5 filter was used as the light source, and the light intensity was calibrated to 100 mW cm^−2^ by a Newport Oriel 91150 V reference cell. Solar Cell Spectral Response Measurement System QE-R3-011 (Enli Technology Co., Ltd., Taiwan) was used to measure the EQE. The light intensity at different wavelength was calibrated with a standard single-crystal Si photovoltaic cell.

### Photo-induced force microscopy (PiFM) and atom force microscopy

The PiFM microscope including AFM microscope is from a VistaScopefrom Molecular Vista, Inc., operated in dynamic mode using commercial gold-coated silicon cantilevers (NCHAu) from Nanosensors. The excitation laser for the PiFM measurements is a LaserTune IR Source from Block Engineering.

### Grazing incident wide-angle X-ray scattering (GIWAXS)

Grazing-incidence wide-angle X-ray scattering (GIWAXS) measurements were conducted at Advanced Light Source (ALS), Lawrence Berkeley National Laboratory, Berkeley, CA at the beamline 7.3.3. Data were acquired at the critical angle (0.130) of the film with a hard X-ray energy of 10 keV. X-ray irradiation time was 30–60 s, dependent on the saturation level of the detector. Beam center was calibrated using AgB powder and the sample-to-detector distance was about 280 mm. The π–π coherence lengths (L) are estimated based on the Scherrer Equation (L = 2πK/FWHM), where K is the shape factor (here we use 0.9), and FWHM is the full width at half maximum of the (010) diffraction peaks.

### Measurement of charge carrier mobilities

The charge carrier mobilities were measured with the device structure of ITO/PEDOT:PSS/active layer/MoO_3_/Ag for hole mobility and ITO/ZnO/active layer/PDINO/Al for electron mobility. The hole and electron mobilities are calculated according to the SCLC method equation: *J* = 9*µ*ɛ_r_ɛ_0_*V*^2^/8*d*^3^, where *J* is the current density, *µ* is the hole or electron mobility, *V* is the internal voltage in the device, ε_r_ is the relative dielectric constant of active layer material, ε_0_ is the permittivity of empty space, and *d* is the thickness of the active layer.

### Transient absorption spectroscopy

A Yb: KGW amplifier (PHAROS, Light Conversion,) supplied laser beams centered at 1030 nm with pulse duration of ~180 fs, pulse repetition rate of 33 kHz, and a maximum pulse energy of 0.3 mJ. The output of the amplifier was split into two streams of pulses. One was used to drive an optical parametric amplifier (ORPHEUS-HP, Light Conversion) to obtain the pump beam. Residual stream was directed into an ultrafast spectroscopic system (HARPIA-TA, Light Conversion) to generate the white light continuum probe beam. In the spectrometer, the pump chopped at 150 Hz frequency was spatially and temporally overlapped with the probe beam on the sample. Pump wavelength was set to 850 nm to selectively excite the polymer acceptors. Excitation energy of the pump pulse was set to 2 μJ/cm^2^ to avoid singlet-singlet annihilation. The film samples for the TA measurements were prepared by spin coating the corresponding materials on quartz plates with 1 mm thick. The TA samples were annealed in nitrogen atmosphere at 120 °C for 5 min prior to measurement.

### Reporting summary

Further information on research design is available in the Nature Research Reporting Summary linked to this article.

## Supplementary information

Supplementary Information

Solar Cells Reporting Summary

## Data Availability

The data that support the findings of this study are available from the corresponding author on request.
