# Supplementary material for: Polymerized small molecular acceptor based all-polymer solar cells with an efficiency of 16.16% via tuning polymer blend morphology by molecular design
Source: Nat Commun. 2021 Sep 6;12:5264. doi: 10.1038/s41467-021-25638-9 (PMC8421507; doi:10.1038/s41467-021-25638-9)
Supplement: Supplementary file 1 — Supplementary Information [file 41467_2021_25638_MOESM1_ESM.pdf]

Supplementary Information

Polymerized small molecular acceptor based all-polymer solar cell with an efficiency of 16.16% *via* modulating polymer blend morphology by molecular design

Jiaqi Du[1,2,#], Ke Hu[2,#], Jinyuan Zhang[1], Lei Meng[1,2], Jiling Yue[3], Indunil Angunawela[4], Honging Yan[5], Shucheng Qin[1,2], Xiaolei Kong[1,2], Zhanjun Zhang[2], Bo Guan[3*], Harald Ade[4*] and Yongfang Li[1,2,6*]*

[1]Beijing National Laboratory for Molecular Sciences, CAS Key Laboratory of Organic Solids, Institute of Chemistry, Chinese Academy of Sciences, 100190 Beijing, China

[2]School of Chemical Science, University of Chinese Academy of Sciences, 100049 Beijing, China

[3]Center for Physiochemical Analysis and Measurement, Institute of Chemistry, Chinese Academy of Sciences, 100190 Beijing, China

[4]Department of Physics and Organic and Carbon Electronics Lab (ORaCEL), North Carolina State University, Raleigh, NC 27695, USA

[5]Department of Chemical Engineering, Stanford University, 443 Via Ortega Stanford, CA 94305-4125

[6]Laboratory of Advanced Optoelectronic Materials, Suzhou Key Laboratory of Novel Semiconductor-Optoelectronics Materials and Devices, College of Chemistry, Chemical Engineering and Materials Science, Soochow University, 215123 Suzhou, Jiangsu, China

[#] J. Du and K. Hu contributed equally to this work.

Supplementary Figures

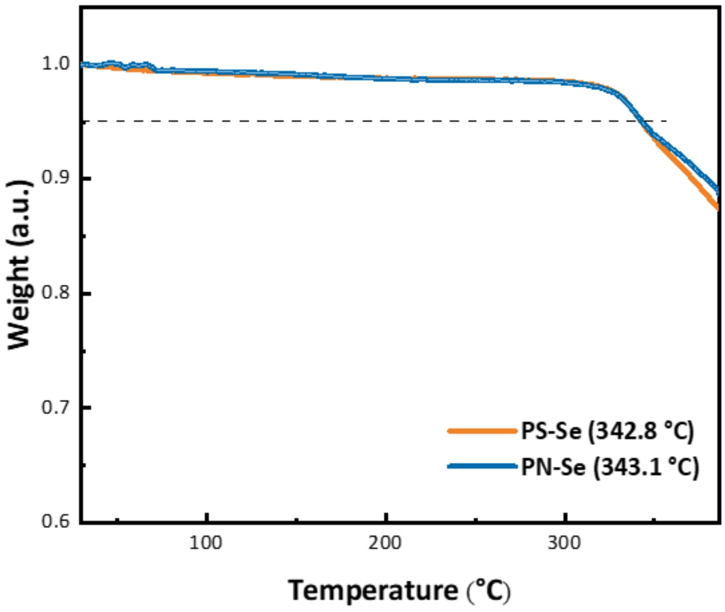

Supplementary Fig. 1. TGA plots of PS-Se and PN-Se with a heating rate of 10 °C min^{-1} under N_2 atmosphere.

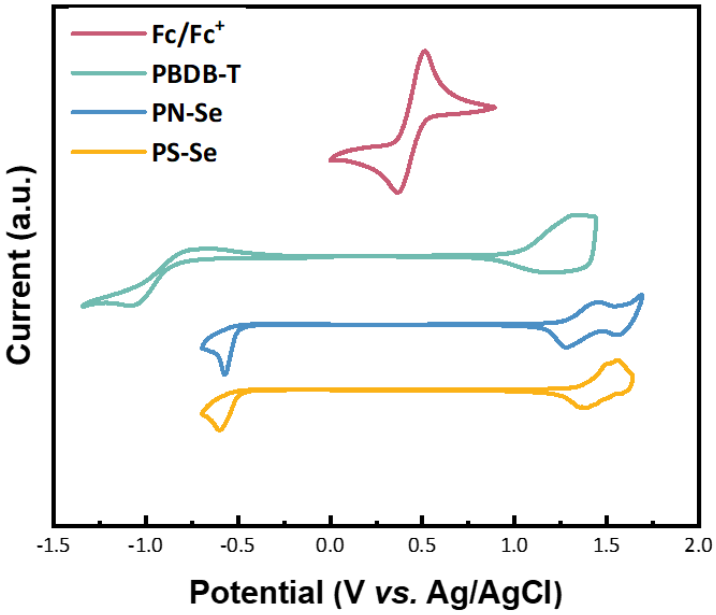

Supplementary Fig. 2. Cyclic voltammograms of polymer donor PBDB-T and PSMA polymer acceptors PS-Se and PN-Se.

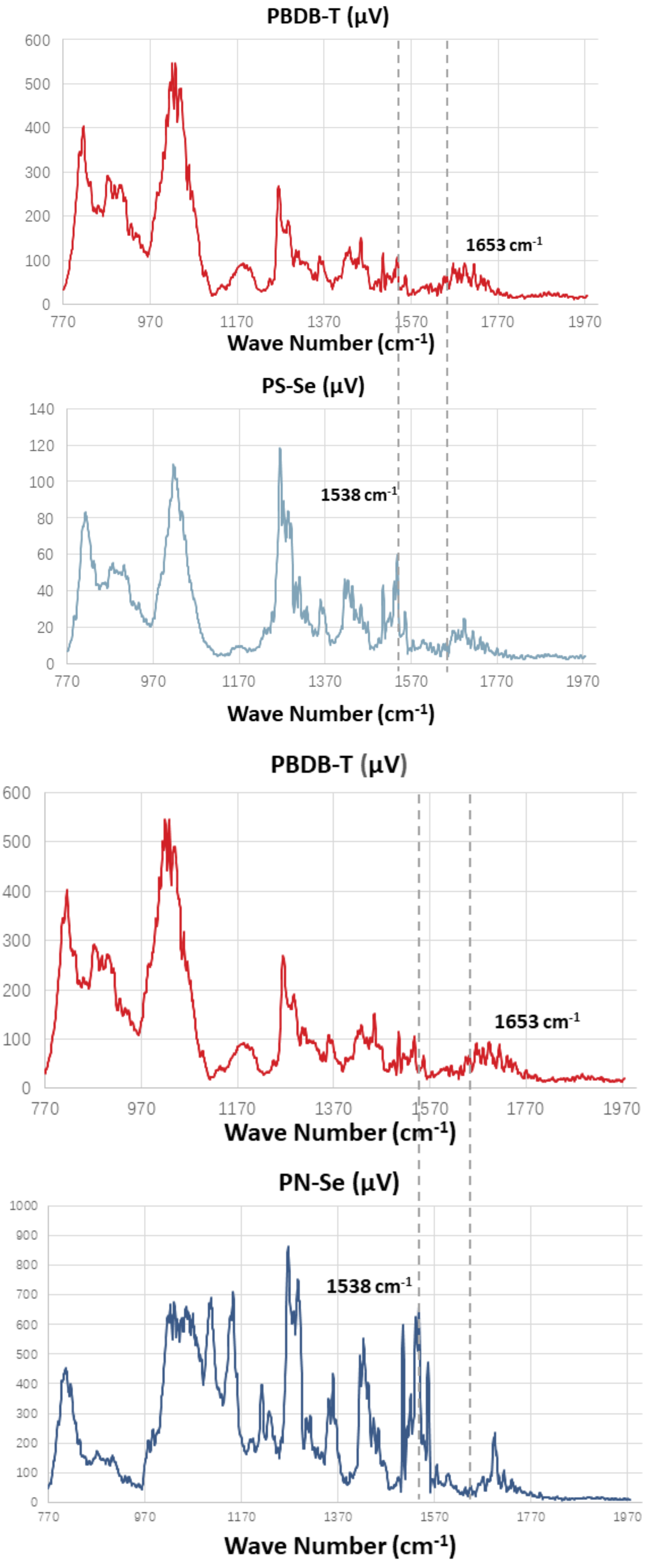

Supplementary Fig. 3. The PiFM infrared (IR) spectra of neat PBDB-T, PS-Se and PN-Se films.

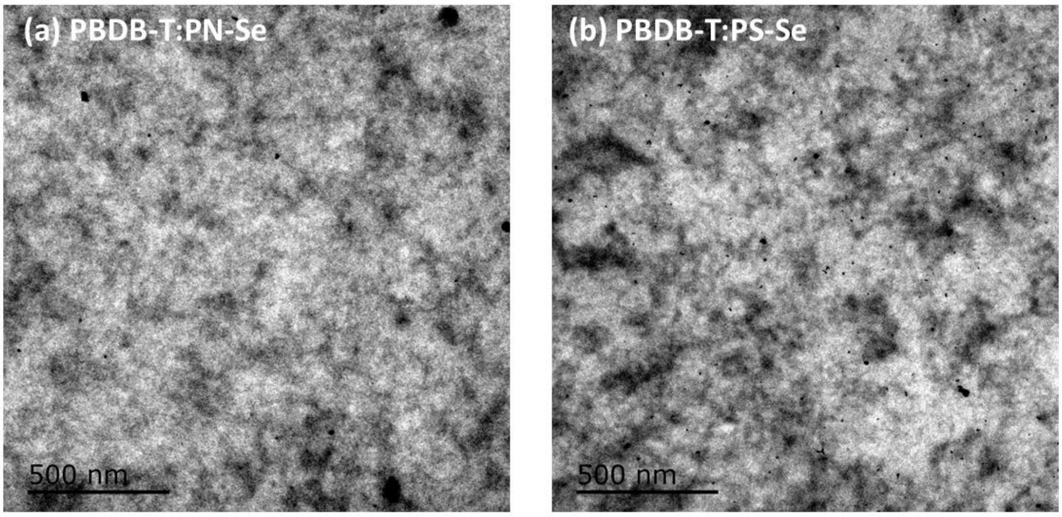

Supplementary Fig. 4. Transmission electron microscopy (TEM) of **a**, PBDB-T:PN-Se and **b**, PBDB-T:PS-Se blended films.

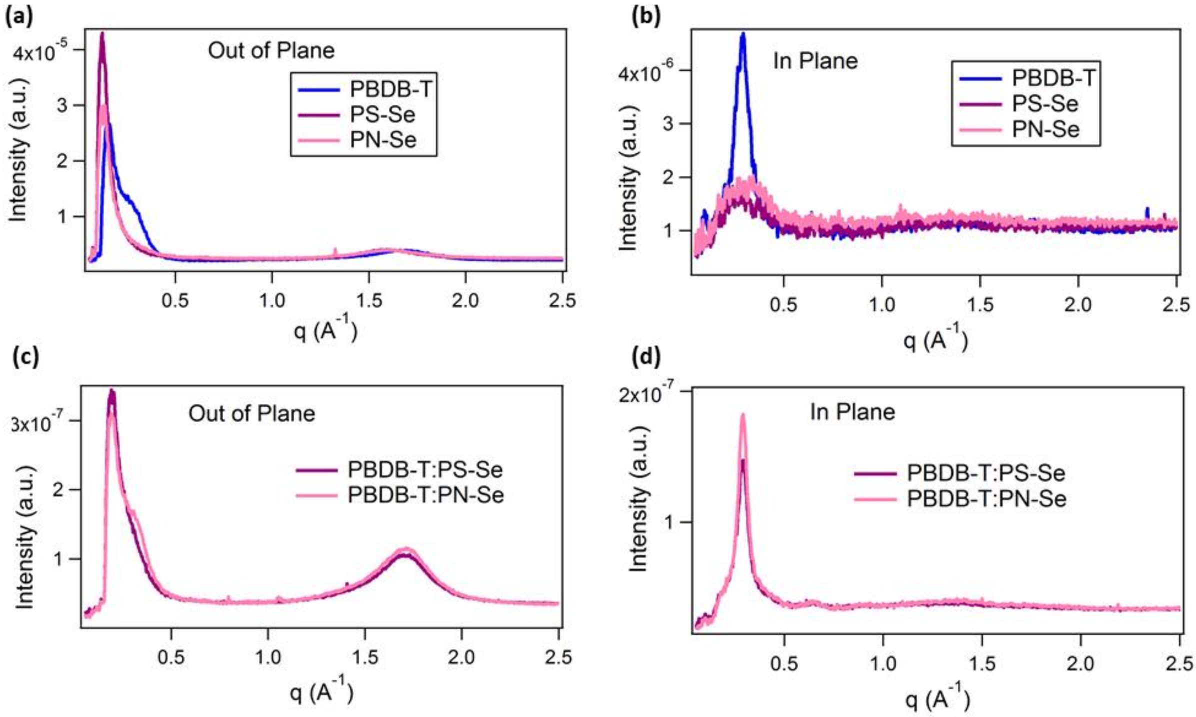

Supplementary Fig. 5. 1D line cuts extracted from the 2D GIWAXS patterns.

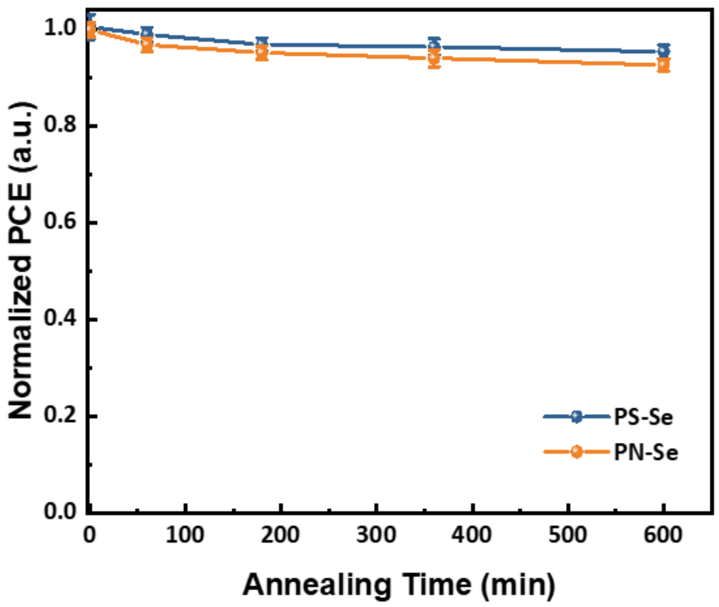

Supplementary Fig. 6. Average normalized PCE of the PS-Se and PN-Se based all-PSCs under 150 ℃ stress for different times.

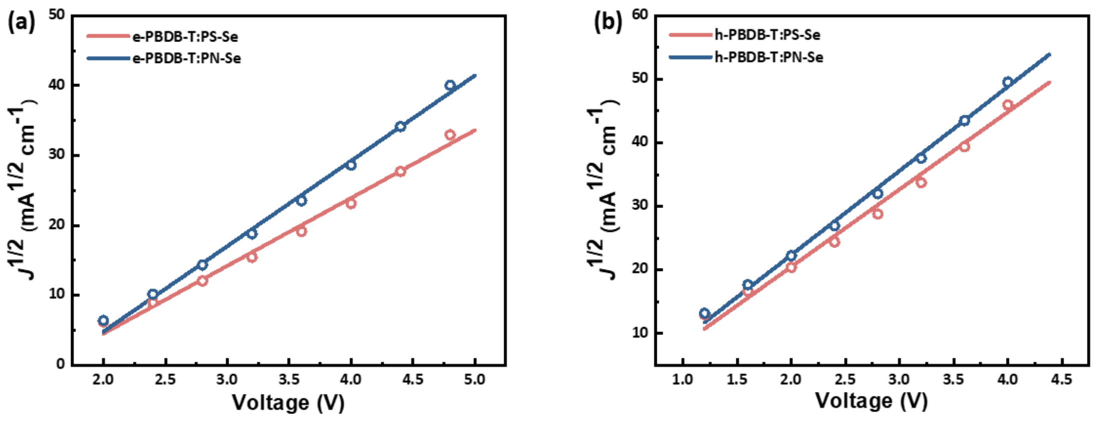

Supplementary Fig. 7. Plots of current density *vs*. voltage of the devices based on different blend active layers for the mobility measurements with SCLC method. (a) electron-only devices for measuring electron mobilities and (b) hole-only devices for measuring hole mobilities.

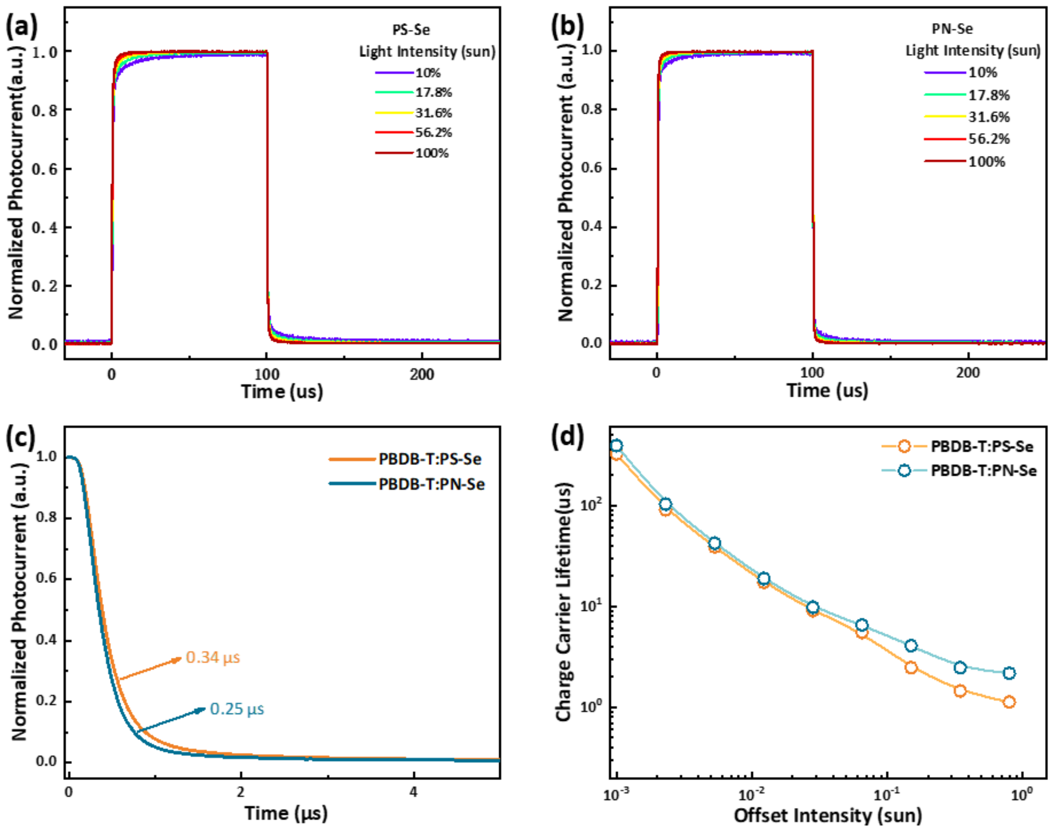

Supplementary Fig. 8. TPC TPV measurements. a, **b**, and **c**, Transient photocurrent of the PS-Se and PN-Se based devices. **d**, The transient photovoltage lifetime spectra of the devices based on PS-Se and PN-Se.

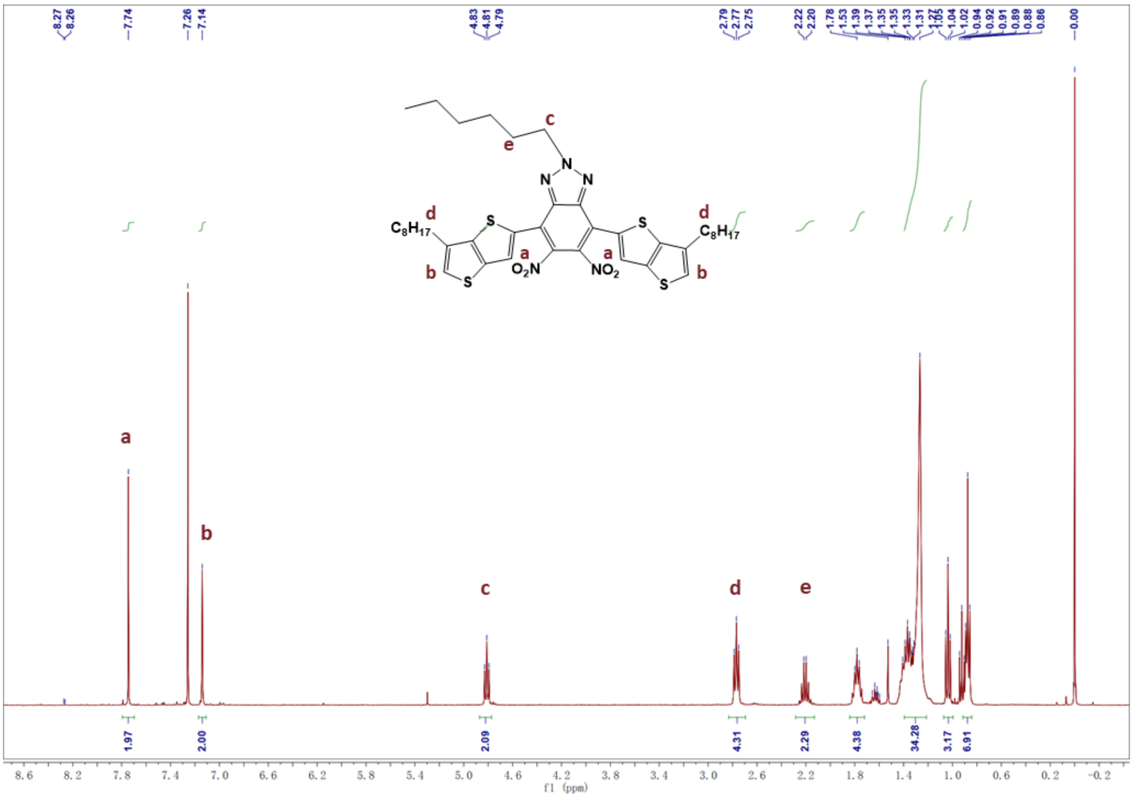

Supplementary Fig. 9. ^{1}H-NMR spectrum of compound **b** in $CDCl_3$.

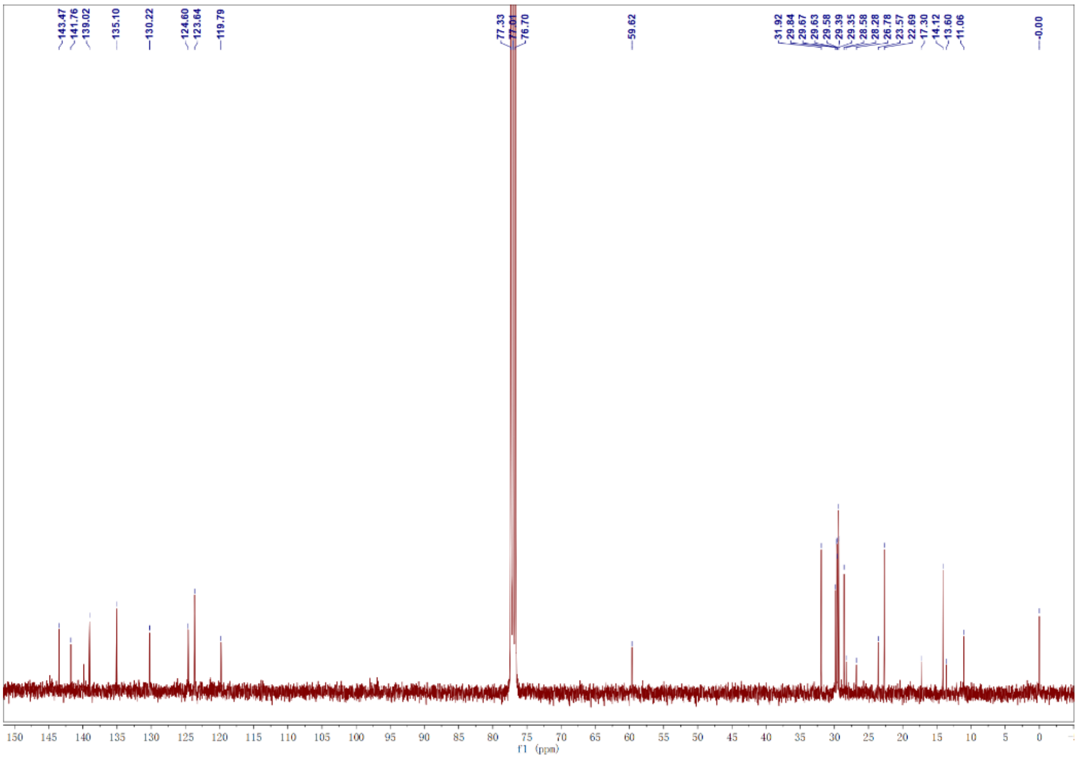

Supplementary Fig. 10. ^{13}C-NMR spectrum of compound **b** in $CDCl_3$.

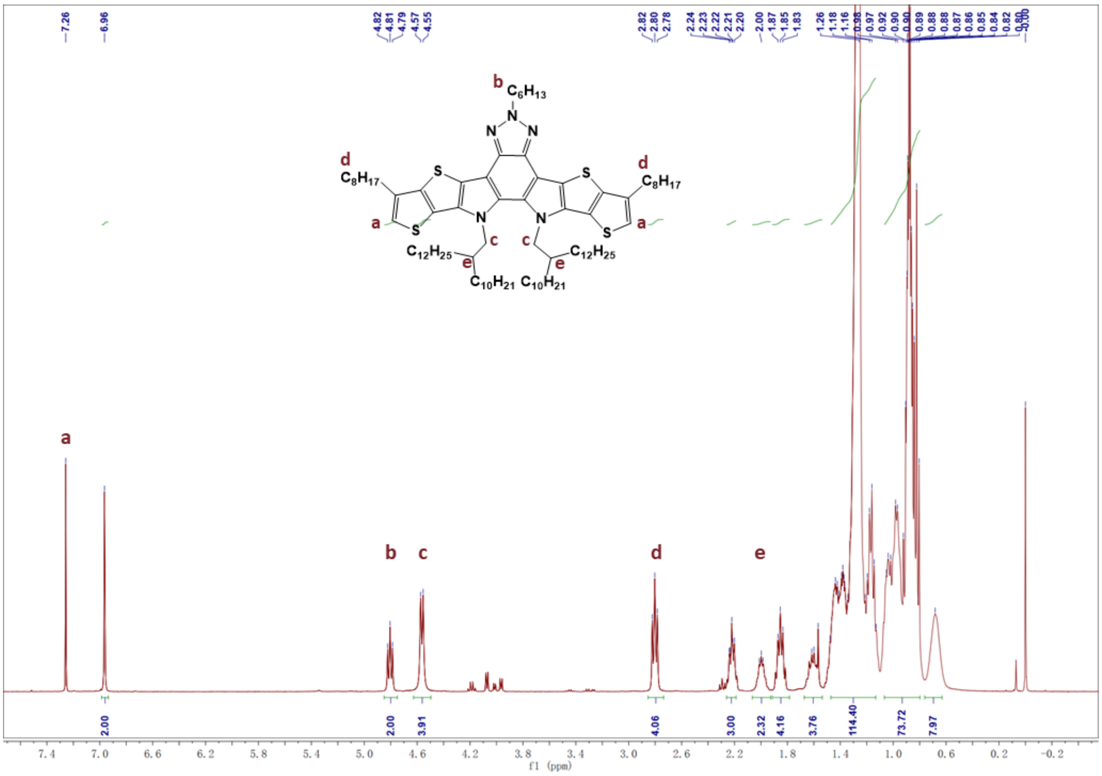

Supplementary Fig. 11. ^{1}H-NMR spectrum of compound **c** in $CDCl_3$.

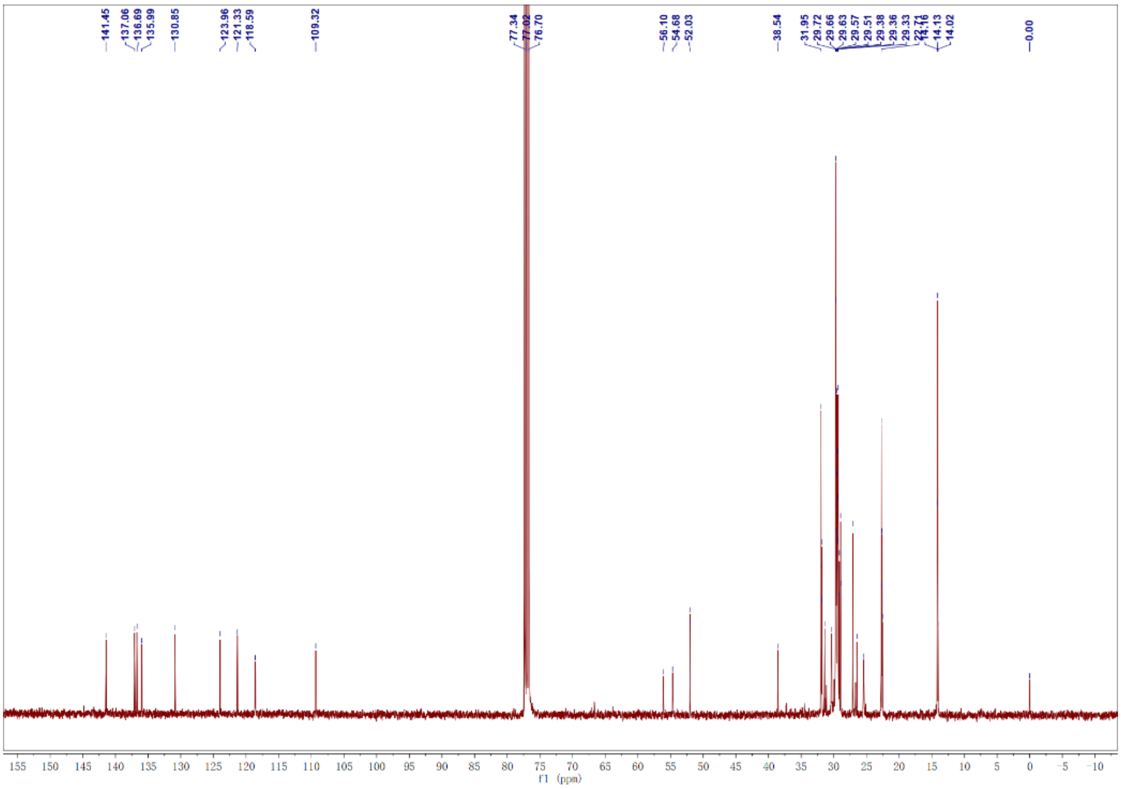

Supplementary Fig. 12. ^{13}C-NMR spectrum of compound **c** in $CDCl_3$.

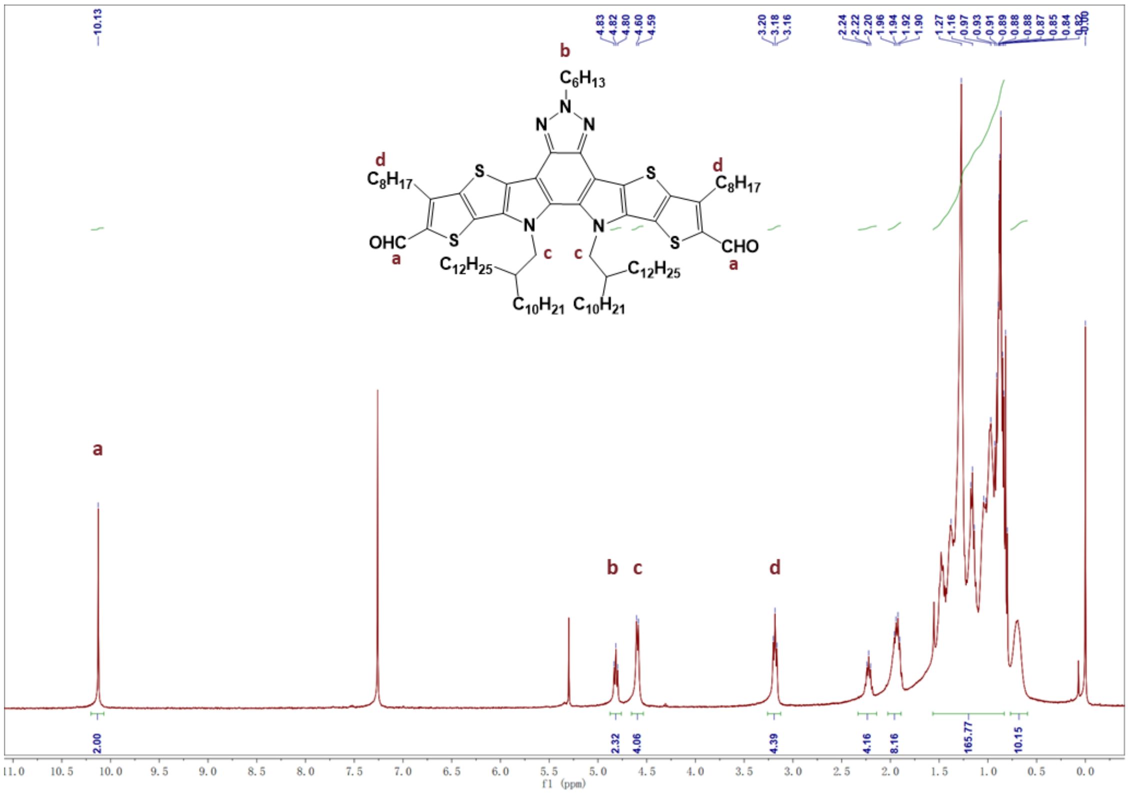

Supplementary Fig. 13. ^{1}H-NMR spectrum of compound TPBN-CHO in $CDCl_3$.

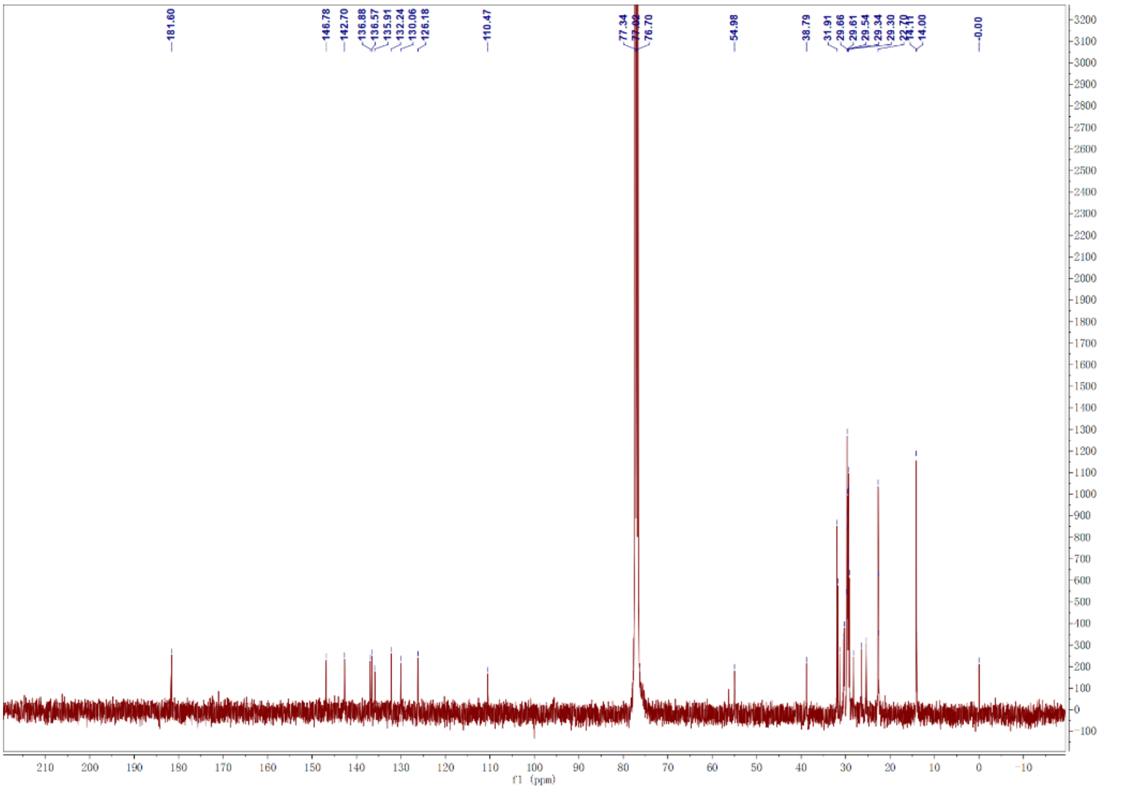

Supplementary Fig. 14. ^{13}C-NMR spectrum of compound TPBN-CHO in $CDCl_3$.

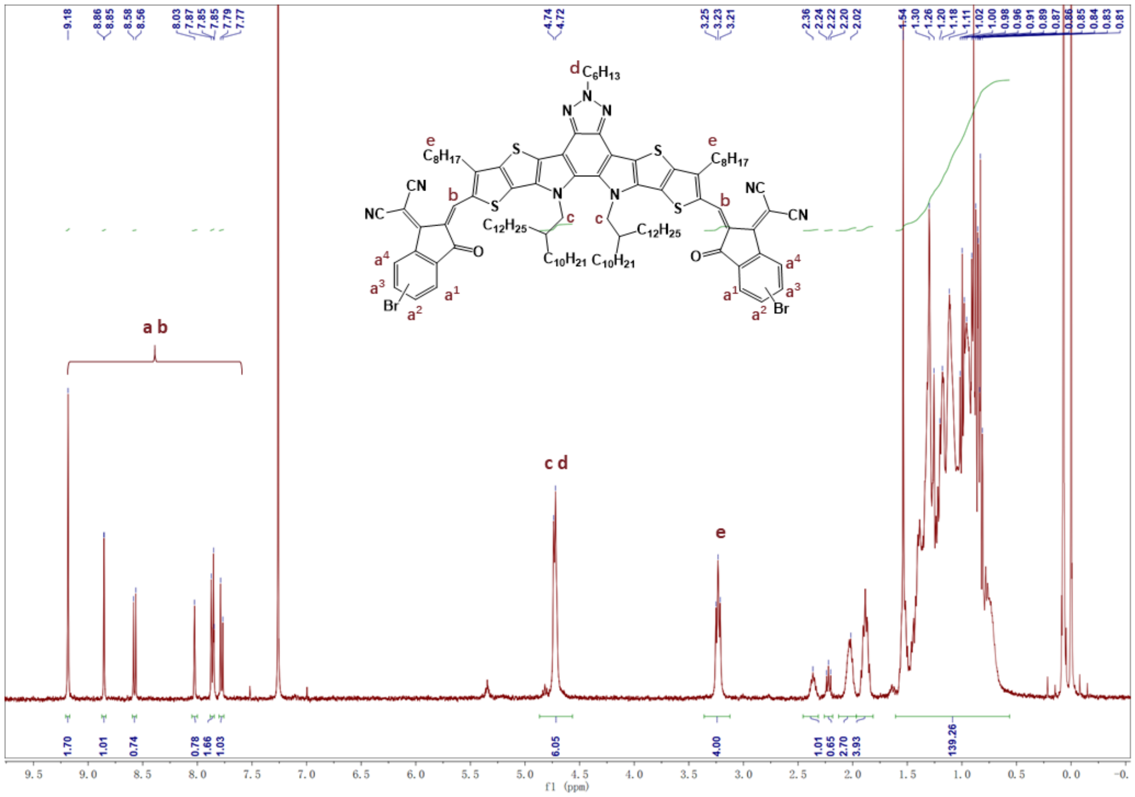

Supplementary Fig. 15. ^{1}H-NMR spectrum of compound TPBN-Br in $CDCl_3$.

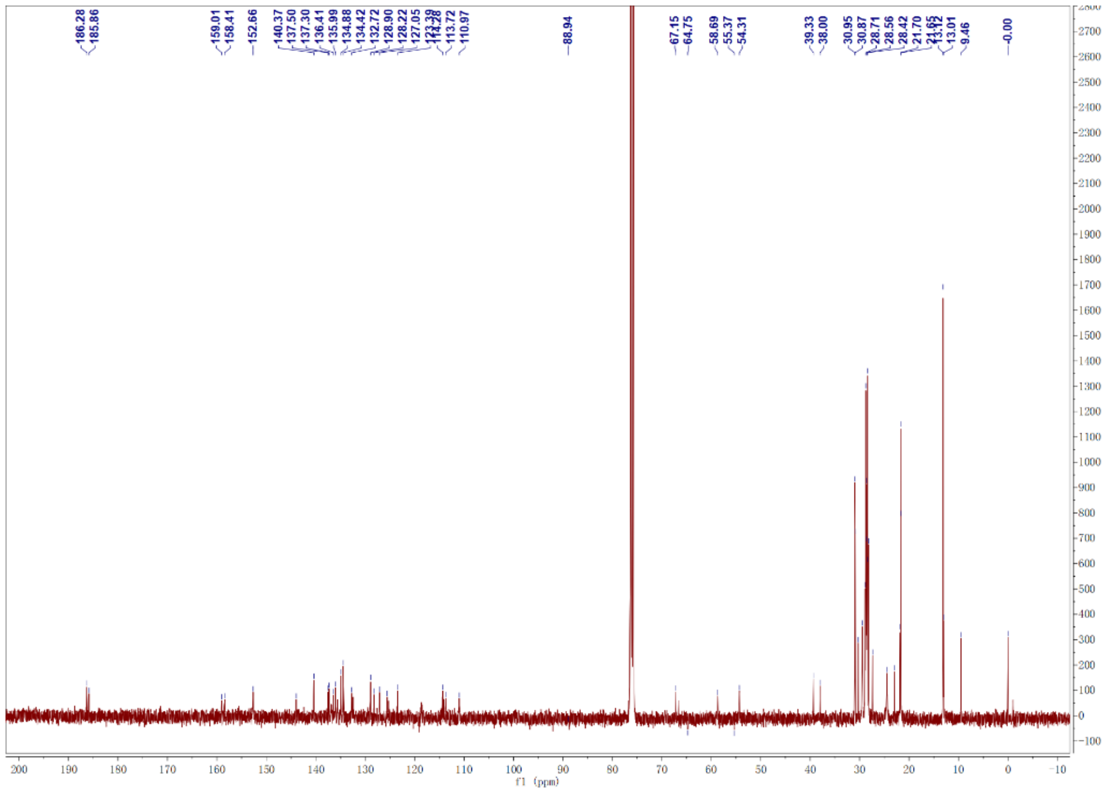

Supplementary Fig. 16. ^{13}C-NMR spectrum of compound TPBN-Br in $CDCl_3$.

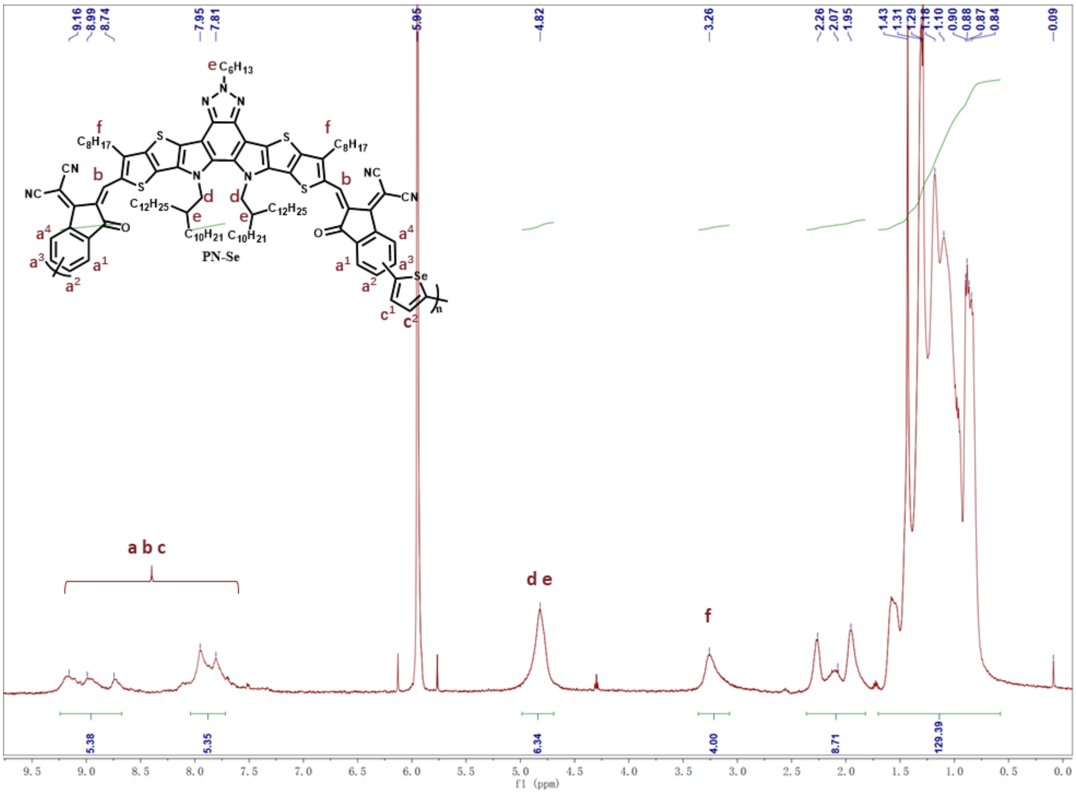

Supplementary Fig. 17. ^{1}H-NMR spectrum of compound PN-Se in 1,1,2,2-tetrachloroethane-d2 at 80 °C.

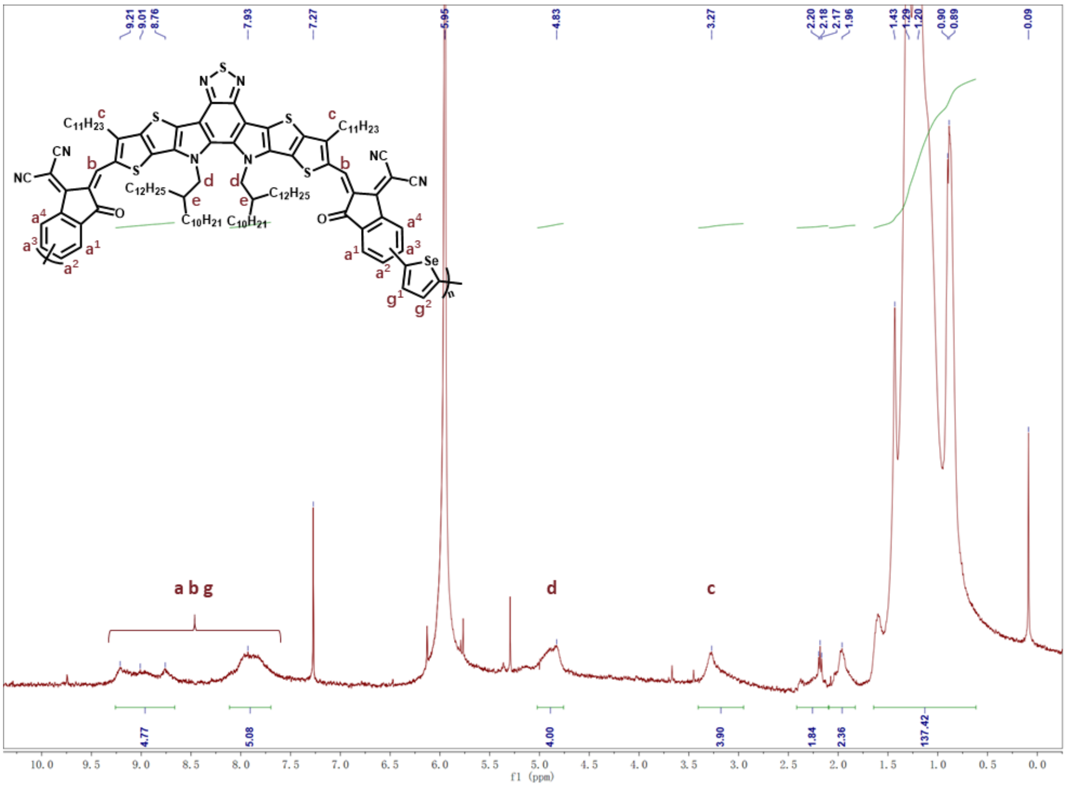

Supplementary Fig. 18. ^{1}H-NMR spectrum of compound PS-Se in 1,1,2,2-tetrachloroethane-d2 at 80 °C

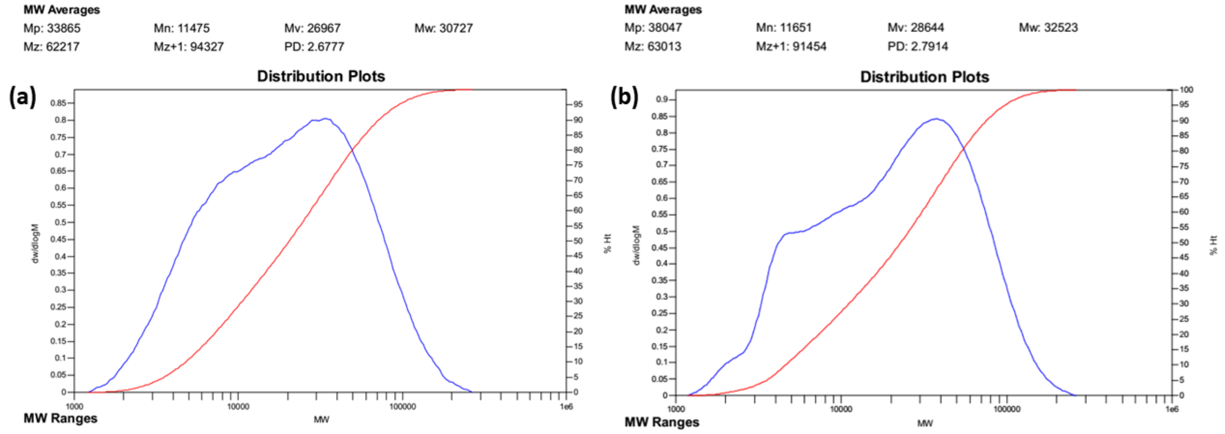

Supplementary Fig. 19. GPC results of **a**, PS-Se and **b**, PN-Se

Supplementary Tables

Supplementary Table 1. Energy levels and optical bandgap of PSMAs.

PSMA	E_{LUMO} (eV) [a]	E_{HOMO} (eV) [a]	E_g (eV)	E_g^{opt} (eV) [b]
PS-Se	-3.88	-5.69	1.81	1.41
PN-Se	-3.85	-5.63	1.78	1.37

[a]Energy levels calculated according to the equation $E_{\mathrm{LUMO/HOMO}} = -e\ (E_{\mathrm{red/ox}} + 4.36)$ (eV). [b]Optical bandgap calculated from the absorption edge of the films: $E_g^{opt} = 1240/\lambda_{\mathrm{edge}}$.

Supplementary Table 2. Contact angles and Flory−Huggins interaction parameters of PSMAs.

$\theta_{\mathbf{Water}}$[a]	$\theta_{\mathbf{Diiodomethane}}$[a]	**γ (mN m^{-1})**	**relative χ[b]**
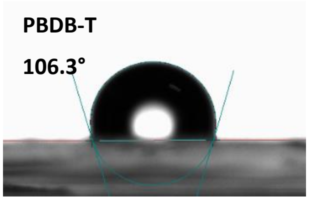	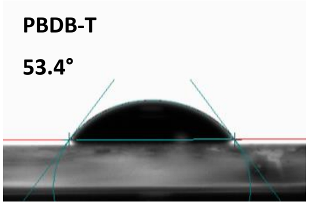	31.12	—
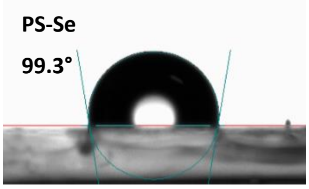	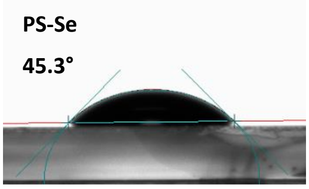	37.63	0.30
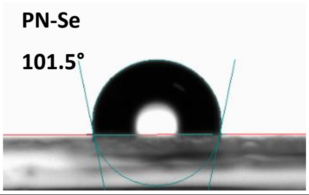	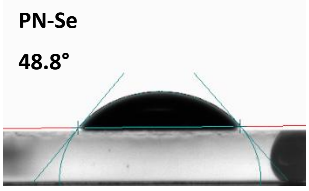	36.23	0.19

[a]The data of contact angels are the average of three tests.

[b]The Flory−Huggins interaction parameter (χ_{da}) are calculated by $\chi = K\left(\sqrt{\gamma_d} - \sqrt{\gamma_a}\right)^2$.

Supplementary Table 3. PBDB-T stacking parameters in the blend.

blend	Out of plane q ($Å^{-1}$)	d (Å)	Δq ($Å^{-1}$)	CCL (Å)	Normalized Integrated Intensity	In plane q ($Å^{-1}$)	d (Å)	Δq ($Å^{-1}$)	CCL (Å)	Normalized Integrated Intensity	g parameter
**PBDB-T:PS-Se**	1.71	3.67	0.22	28.6	0.85	0.29	21.7	0.05	126	0.75	14.3
**PBDB-T:PN-Se**	1.71	3.67	0.23	27.3	1.00	0.29	21.7	0.05	126	1.00	14.6

Supplementary Table 4. Acceptor stacking parameters in the blend.

blend	Out of plane q ($Å^{-1}$)	d (Å)	Δq ($Å^{-1}$)	CCL (Å)	Normalized Integrated Intensity	In plane q ($Å^{-1}$)	d (Å)	Δq ($Å^{-1}$)	CCL (Å)	Normalized Integrated Intensity	g parameter
**PBDB-T:PS-Se**	1.65	3.81	0.54	11.6	0.91	0.28	22.4	0.20	31.4	0.87	22.8
**PBDB-T:PN-Se**	1.65	3.81	0.54	11.6	1.00	0.28	22.4	0.20	31.4	1.00	22.8

Supplementary Table 5. Performance parameters of the all-PSCs based on PBDB-T:PSMA (1.5:1, wt/wt, annealing at 120℃) with different concentration of additives under illumination of AM 1.5G, 100 mW cm^{-2}.

	Additive (vol %)	V_{oc} (V)	J_{sc} (mA cm^{-2})	FF	PCE (%)
PBDB-T:PS-Se	No	0.868	21.75	0.533	10.06
	1	0.869	23.06	0.650	13.03
	1.5	0.874	23.27	0.680	13.83
	2	0.883	21.53	0.715	13.59
PBDB-T:PN-Se	No	0.903	22.56	0.581	11.83
	1	0.902	24.63	0.709	15.75
	1.5	0.907	24.82	0.718	16.16
	2	0.908	24.65	0.702	15.71

Supplementary Table 6. Performance parameters of all-PSCs based on PBDB-T:PSMA (1.5:1, wt/wt, CN: 1.5 vol%) with different annealing temperature.

	Annealing temperature (°C)	V_{oc} (V)	J_{sc} (mA cm^{-2})	FF	PCE (%)
PBDB-T:PS-Se	110	0.874	22.98	0.681	13.68
	120	0.874	23.27	0.680	13.83
	140	0.869	22.30	0.678	13.14
PBDB-T:PN-Se	110	0.906	24.76	0.712	15.97
	120	0.907	24.82	0.714	16.16
	140	0.899	24.56	0.711	15.70

Supplementary Table 7. Summary of the molecular weights of PS-Se and PN-Se and the PCEs of the all-PSCs with PBDB-T polymer donor and the PSMA polymer acceptors with different molecular weight.

Polymer	PS-Se		PN-Se	
Batches	M_n (kDa)	PCE_{max} (%)	M_n (kDa)	PCE_{max} (%)
Batch1	8.8	12.7	9.5	15.6
Batch2	10.2	13.5	11.7	16.1
Batch3	11.5	13.8	12.2	16.0
Batch4	18.7	No[a]	17.5	15.3

[a] The batch 4 of PS-Se is not soluble in chloroform.

Supplementary Table 8. Hole and electron mobilities obtained from SCLC measurements.

Blend	$\mu_{electron}$ (×10^{-4} cm^2 V^{-1} s^{-1})[a]	μ_{hole} (×10^{-4} cm^2 V^{-1} s^{-1})[a]
PBDB-T:PS-Se	4.84±0.48	6.89±0.52
PBDB-T:PN-Se	6.87±0.50	7.92±0.49

[a] The average parameters were calculated from ten devices.

Supplementary method

Synthesis of 2-hexyl-4,7-bis(6-octylthieno[3,2-b]thiophen-2-yl)-5,6-dinitro-2H-benzo[d][1,2,3]triazole (b)

Tributyl(6-octylthieno[3,2-b]thiophen-2-yl)stannane (3.25 g, 6 mmol), 4,7-dibromo-2-hexyl-5,6-dinitro-2H-benzo[d][1,2,3]triazole (1.13 g, 2.5 mmol) and $Pd(PPh_3)Cl_2$ (0.73 g, 0.10 mmol) were dissolved in 110 mL of dry toluene and stirred at 110 °C overnight. The reaction mixture was allowed to cool to room temperature and was quenched with water. It is then extracted with an aqueous solution containing dilute hydrochloric acid. The crude product was purified on silica gel chromatography using petroleum ether/CH_2Cl_2 (3:1, v/v) to give compound **b** (1.45 g) as an orange solid in a yield of 75%. ^{1}H NMR (400 MHz, $CDCl_3$). δ 7.74 (s, 2H), 7.14 (s, 2H), 4.81 (t, J = 7.2 Hz, 2H), 2.77 (t, J = 7.6 Hz, 4H), 2.28 – 2.13 (m, 2H), 1.84 – 1.72 (m, 4H), 1.39 – 1.21 (m, 34H), 1.07 – 1.00 (m, 3H), 0.91 – 0.84 (m, 7H). ^{13}C NMR (101 MHz, $CDCl_3$) δ 141.45, 137.06, 136.69, 135.99, 130.85, 123.96, 121.33, 118.59, 109.32, 77.34, 77.02, 76.70, 56.10, 54.68, 52.03, 38.54, 31.95, 31.91, 31.86, 31.81, 31.39, 30.39, 29.72, 29.66, 29.63, 29.60, 29.57, 29.51, 29.45, 29.43, 29.41, 29.38, 29.36, 29.33, 29.24, 29.15, 28.95, 28.89, 27.10, 26.46, 25.42, 22.71, 22.67, 22.62, 22.52, 14.16, 14.13, 14.02. HRMS(TOF) m/z calcd. for $[M]^+$ $C_{40}H_{51}N_5O_4S_4$: 793.28, found 793.3113.

Synthesis of 6-hexyl-3, 9-dioctyl-12, 13-bis(2-octyldodecyl)-12, 13-dihydro-6H-thieno[2'',3'':4',5']-thieno[2',3':4,5]pyrrolo[3,2-g]thieno[2',3':4,5]thieno[3,2-b][1,2,3]triazolo[4,5-e]indole (c)

Compound **b** (1.49 g, 1.88 mmol) and triethyl phosphite (20 mL) were dissolved in dichlorobenzene (o-DCB, 10 mL) under nitrogen. The reaction was carried out at 180 °C for 10

hours, after extracted with dichloromethane and water, dried with magnesium sulfate anhydrous, and then evaporated. The crude product was obtained directly used as the next reaction. The crude product, 11-(bromomethyl)tricosane (2.36 g, 5.64 mmol), potassium iodide (0.33 g, 2mmol) and potassium carbonate (1.38 g, 10 mmol) and DMF (80 mL) was taken in a 250 ml one-neck flask. Stir at 100 ° C overnight under nitrogen. Cool to room temperature and spin dry with ethyl acetate and water. Further purification by column chromatography using dichloromethane / petroleum ether (1/10, v / v) as eluent to afford yellow adhesive liquid **c** (1.13 g, 43% yield). ^{1}H NMR (400 MHz, $CDCl_3$) δ 6.96 (s, 2H), 4.80 (t, *J* = 7.5 Hz, 2H), 4.56 (d, *J* = 7.6 Hz, 4H), 2.80 (t, *J* = 7.7 Hz, 4H), 2.26 – 2.19 (m, 3H), 2.07 – 1.93 (m, 2H), 1.92 – 1.78 (m, 4H), 1.67 – 1.54 (m, 4H), 1.47 – 1.13 (m, 114H), 1.07 – 0.80 (m, 74H), 0.76 – 0.63 (m, 8H). ^{13}C NMR (101 MHz, $CDCl_3$) δ 143.47, 141.76, 139.02, 135.10, 130.22, 124.60, 123.64, 119.79, 77.33, 77.01, 76.70, 59.62, 31.92, 29.84, 29.67, 29.63, 29.58, 29.39, 29.35, 28.58, 28.28, 26.78, 23.57, 22.69, 17.30, 14.12, 13.60, 11.06. HRMS(TOF) m/z calcd. for $[M]^+$ $C_{88}H_{147}N_5S_4$: 1402.05, found 1402.0547.

Synthesis of 6-hexyl-3, 9-dioctyl-12, 13-bis(2-octyldodecyl)-12, 13-dihydro-6H-thieno[2'',3'':4',5']-thieno[2',3':4,5]pyrrolo[3,2-g]thieno[2',3':4,5]thieno[3,2-b][1,2,3]triazolo[4,5-e]indole-2,10- dicarbaldehyde (TPBN-CHO)

Compound **TPBN-CHO** was synthesized by Vilsmeier-Haack reaction. $POCl_3$ (2.00 mL, 22 mmol) was added dropwise into 4 mL DMF under nitrogen at 0°C. After stirred for 1h, the forming Vilsmeier reagent was added into 10 mL chloroform solution of **c** (1.13 g, 0.81 mmol). The mixture was stirred at ice bath for 30 min and then heated to 60°C for 24 h. After cooling to room temperature, water was added to quench the reaction. The mixture was extracted with CH_2Cl_2 for three times and the combined organic phase was washed with saturated salt solution. The final

organic phase was dried over anhydrous $MgSO_4$. After filtration, the solvent was removed under reduced pressure. The crude product was purified on silica gel chromatography using petroleum ether/ CH_2Cl_2 (1:1, v/v) to give compound **TPBN-CHO** as an orange oil (0.86 g, 73%). ^{1}H NMR (400 MHz, $CDCl_3$). δ 10.13 (s, 2H), 4.82 (t, *J* = 7.5 Hz, 2H), 4.59 (d, *J* = 7.5 Hz, 4H), 3.18 (t, *J* = 7.7 Hz, 4H), 2.33 – 2.14 (m, 4H), 2.03 – 1.89 (m, 8H), 1.56 – 0.60 (m, 176H). ^{13}C NMR (101 MHz, $CDCl_3$) δ 181.60, 146.78, 142.70, 136.88, 136.57, 135.91, 132.24, 130.06, 126.18, 110.47, 77.34, 77.02, 76.70, 54.98, 38.79, 31.91, 31.77, 31.33, 30.43, 30.26, 29.76, 29.66, 29.61, 29.54, 29.50, 29.41, 29.34, 29.30, 29.14, 28.23, 26.41, 25.36, 22.70, 22.60, 22.50, 14.11, 14.00. HRMS(TOF) m/z calcd. for $[M]^+$ $C_{90}H_{147}N_5O_2S_4$: 1458.04, found 1458.0428.

Synthesis of TPBN-Br

Compound **TPBN-CHO** (210.1 mg, 0.15 mmol), IC-Br (162.6 mg, 0.6 mmol), pyridine (1 mL) and chloroform (30 mL) were dissolved in a round bottom flask under nitrogen. The mixture was stirred at 35 °C overnight. After cooling to room temperature, the mixture was poured into methanol and filtered. The residue was purified with column chromatography on silica gel using dichloromethane/petroleum ether (1/1, v/v) as the eluent to give a dark blue solid **TPBN-Br** (226.2 mg, 80% yield). ^{1}H NMR (400 MHz, $CDCl_3$) δ 9.18 (s, 2H), 8.85 (d, *J* = 1.3 Hz, 1H), 8.57 (d, *J* = 8.5 Hz, 1H), 8.03 (s, 1H), 7.86 (t, *J* = 5.2 Hz, 2H), 7.78 (d, *J* = 7.9 Hz, 1H), 4.73 (d, 6H), 3.36 – 3.12 (m, 4H), 2.45 – 2.31 (m, 1H), 2.26 – 2.18 (m, 1H), 2.13 – 1.97 (m, 3H), 1.97 – 1.81 (m, 4H), 1.61 – 0.56 (m, 139H). HRMS(TOF) m/z calcd. for $[M]^+$ $C_{114}H_{153}Br_2N_9O_2S_4$: 1967.94, found 1967.9431.

Synthesis of PN-Se

2,5-bis(trimethylstannyl)selenophene monomer (45.67 mg, 0.1 mmol) and bromide **TPBN-Br**

(186.73 mg, 0.1 mmol) were dissolved in toluene (10 mL). Pd $(PPh_3)_4$ (5 mg) was added into the mixtures after being flushed with argon for five minutes. Then, the reaction mixtures were purged with argon for another 15 min. The reactions were stirred at 110 °C for 24 h. The polymers were precipitated in methanol (100 mL) and filtrated. The dried precipitates were purified by Soxhlet extractor and then subjected to Soxhlet extractions with methanol, hexane, acetone, and chloroform each for 10 hours, respectively. Then the chloroform fraction was concentrated and precipitated with methanol, the black solids **PN-Se** was obtained. (125.46 mg, 68% yield). ^{1}H NMR (500 MHz, $CDCl_2CDCl_2$) δ 9.24 – 8.67 (m, 5H), 8.04 – 7.72 (m, 5H), 4.82 (br, 6H), 3.26 (br, 4H), 2.36 – 1.82 (m, 9H), 1.68 – 0.62 (m, 129H). M_n = 11.7 KDa; M_w/M_n =2.79.

Synthesis of PS-Se

2,5-bis(trimethylstannyl)selenophene monomer (45.67 g, 0.1 mmol) and bromide **TPBS-Br** (198.66 g, 0.1 mmol) were dissolved in toluene (10 mL). Pd $(PPh_3)_4$ (5 mg) was added into the mixtures after being flushed with argon for five minutes. Then, the reaction mixtures were purged with argon for another 15 min. The reactions were stirred at 110 °C for 24 h. The polymers were precipitated in methanol (100 mL) and filtrated. The dried precipitates were purified by Soxhlet extractor and then subjected to Soxhlet extractions with methanol, hexane, acetone, and chloroform each for 10 hours, respectively. Then the chloroform fraction was concentrated and precipitated with methanol, the black solids **PS-Se** was obtained. (139.16 mg, 70% yield). ^{1}H NMR (500 MHz, $CDCl_2CDCl_2$) δ 9.26 – 8.66 (m, 5H), 7.93 (br, 5H), 4.83 (br, 4H), 3.27 (br, 4H), 2.42 – 2.10 (m, 2H), 2.09 – 1.83 (m, 2H), 1.64 – 0.62 (m, 137H). M_n = 11.5 KDa; M_w/M_n =2.68.
